# Five New Guanacastane-Type Diterpenes from Cultures of the Fungus *Psathyrella candolleana*

**DOI:** 10.1007/s13659-014-0020-8

**Published:** 2014-05-08

**Authors:** Xia Yin, Tao Feng, Zheng-Hui Li, Ying Leng, Ji-Kai Liu

**Affiliations:** 1 State Key Laboratory of Phytochemistry and Plant Resources in West China, Kunming Institute of Botany, Chinese Academy of Sciences, Kunming, 650201 People’s Republic of China; 2 University of Chinese Academy of Sciences, Beijing, 100049 People’s Republic of China; 3 Shanghai Institute of Materia Medica, Chinese Academy of Sciences, Shanghai, 201203 People’s Republic of China

**Keywords:** *Psathyrella candolleana*, Psathyrellaceae diterpenes, Guanacastepenes P–T, 11*β*-HSD1

## Abstract

Five new guanacastane-type diterpenes, named guanacastepenes P–T (**1**–**5**), were isolated from cultures of the fungus *Psathyrella candolleana.* Their structures were elucidated on the basis of extensive spectroscopic methods. All of the compounds were tested for their 11*β*-hydroxysteroid dehydrogenase (11*β*-HSD1) inhibitory activity. Compound **3** exhibited inhibitory activity against both human and mouse isozymes of 11*β*-HSD1 with IC_50_ values of 6.2 and 13.9 μM, respectively.

**Electronic supplementary material:**

The online version of this article (doi:10.1007/s13659-014-0020-8) contains supplementary material, which is available to authorized users.

## Introduction

**Fig. 1:**
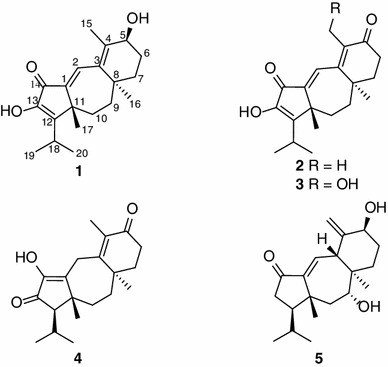
Structures of compounds (**1**–**5**)

Guanacastepene A, was the first member of the guanacastane family isolated from an unidentified endophytic fungus (CR 115) [1]. This was followed by isolation of guanacastepenes B–O from the same fungus by the same group [2]. Heptemerones A–G were later isolated from cultures of *Coprinus heptemerus* [3, 4], as well as radianspenes A–M from *Coprinus radians* [5], which attracted interest of the guanacastanes in the synthetic organic community [6–18]. The total synthesis of guanacastepenes with a novel 5/7/6 ring system was considered a challenging synthetic target [19], whereas synthesis of analogues offered the best prospects for capitalizing on the promising antibiotic activity, as well as avoiding the side effect of hemolytic activity against human red blood cells [10, 20]. However, no other bioactivities, except for antibiotic effects were reported for this type of diterpenes. As a part of a search for naturally occurring secondary metabolites with diverse structures from higher fungi in China, investigations of chemical components from *Psathyrella candolleana* cultures were carried out, which led to the isolation of a series of new guanacastane-type diterpenes, guanacastepenes P–T (**1**–**5**, Fig. 1). Their structures were elucidated by means of spectroscopic methods. These compounds shared a larger conjugated system including an *α*,*β*-unsaturated ketone moiety in the highly oxygenated five-member ring system comparing to the known guanacastane-type diterpenes. All of these compounds were evaluated for their cytotoxic and anti-herpes simplex viruses (HSV) activities, while compounds **1**, **3**, and **5** were evaluated for inhibitory activities against one isozyme of 11*β*-hydroxysteroid dehydrogenases (11*β*-HSD1).

## Results and Discussion

**Fig. 2:**
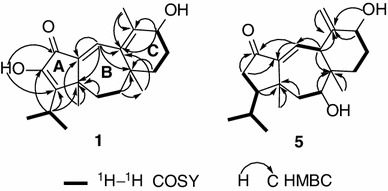
^1^H–^1^H COSY and HMBC correlations for **1** and **5**

Guanacastepene P (**1**) was obtained as a light yellow oil. Its molecular formula C_20_H_28_O_3_ was established from the positive ion HRESIMS ([M+Na]^+^, 339.1930), indicating 7 degrees of unsaturation. The ^13^C NMR (DEPT) spectrum suggested 20 carbon resonances (Table 2), classified as five methyl groups, four aliphatic methylenes, three methines (one olefinic and one oxygenated), and eight quaternary carbons (one carbonyl and five olefinic). Thus, four degrees of unsaturation were accounted for by the three double bonds and the one carbonyl group, while the remaining three degrees of unsaturation suggested that compound **1** should possess a three-ring system. Further inspection of the ^1^H NMR spectrum and ^1^H–^1^H COSY correlations (Fig. 2) resulted in the deduction of an isopropyl group (Me-19–H-18–Me-20), two connected methylene groups (H_2_-9–H_2_-10), a chain of CH_2_–CH_2_–CH–OH (H_2_-7–H_2_-6–H-5–OH), an isolated olefinic hydrogen (H-2), and three isolated methyl groups (H-15, H-16 and H-17).

**Fig. 3:**
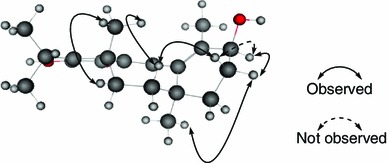
Key ROESY correlations of **1**

Connectivity among the above mentioned fragments and the contribution of the three-ring system were established by analysing ^1^H, ^13^C long-range correlations extracted from HMBC experiments (Fig. 2). The correlations of H-18 to C-11, C-12 and C-13 and HO-13 to C-12 and C-14 established the connection of C-11–C-12–C-13–C-14, while the correlations of H-17 to C-11, C-12 and C-1, and H-2 to C-1 and C-11 indicated the presence of a five-membered ring A. Subsequently, the correlations of H-9 to C-8 and H-10 to C-11 supported the connection of C-8–C-9–C-10–C-11. Besides, correlations of H-16 to C-8 and C-3, as well as H-2 to C-8 were evident for a seven-membered ring B. The last ring C and the links of remaining blocks were clearly shown by HMBC correlations of H-15 to C-3, C-4 and C-5, H-5 to C-4 and C-6, H-6 to C-4 and C-5, and H-7 to C-8 and C-3. Finally, compound **1** possessed a backbone of a 5/7/6 ring system related to that of guanacastepene A [1]. Biogenetically, the stereoconfigurations of methyls of Me-16 and Me-17 were *α* and *β* oriented, respectively [4]. In the ROESY spectrum (Fig. 3), correlations between H-17, H-9*β* and H-10*β* were observed, suggesting both were *β* oriented, while correlations of H-5/H-6*α*, and H-6*α*/H-16 indicated that H-5 was *α* oriented.

Guanacastepene Q (**2**), purified as a light yellow oil, had a molecular formula of C_20_H_26_O_3_ according to its HRESIMS at *m*/*z* 315.1962 ([M+H]^+^). The ^13^C NMR (Table 2) spectroscopic data were similar to those of compound **1**. The main difference was that an oxygenated methine at *δ*_C_ 70.3 (C-5) in **1** was replaced by a keto carbonyl group at *δ*_C_ 197.5 in **2**, constructing a 3,4-unsaturated-5-keto moiety, which led to the downfield shift of C-3 (∆*δ* 20.8 ppm) and C-6 (∆*δ* 4.4 ppm), and the upfield shift of C-4 (∆*δ* 6.4 ppm) in compound **2**, consistent with the HMBC correlations of H-6, H-7 and H-15 to C-5. Detailed analysis of 1D and 2D NMR data (HSQC, HMBC, ^1^H–^1^H COSY, ROESY) suggested that the other parts of **2** were the same as those of **1**. Therefore, compound **2** was established as shown.

Guanacastepene R (**3**), a light yellow oil, had a molecular formula of C_20_H_26_O_4_ on the basis of its HRESIMS at *m*/*z* 353.1737 ([M+Na]^+^), 16 mass units higher than that of **2**. The 1D NMR spectroscopic data (Tables 1 and 2) were quite similar to those of **2**, except that the signals for a methyl group in **2** were replaced by the signals for an oxygenated methylene group, which was confirmed by the HMBC correlations of *δ*_H_ 3.62 (1H, d, *J* = 7.0 Hz, OH-15) to *δ*_C_ 56.4 (t, C-15) and *δ*_C_ 136.3 (s, C-4). Careful analysis of the spectroscopic data finally established the structure of **3** as guanacastepene R, as shown.

**Table 1:** ^1^H NMR [*δ*_H_ (mult, *J* (Hz))] spectroscopic data (400 MHz) for guanacastepenes P–T (**1**–**5**)

No.	**1** ^a^	**2** ^a^	**3** ^a^	**4** ^a^	**5** ^b^
1					
2	6.90, br. s	6.96, br. s	7.17, s	3.50, d (19.0) 3.66, d (19.0)	6.85, d (4.1)
3					3.43, br. s
5	4.02, m				4.42, m
6*α* 6*β*	1.91, m 1.75, m	2.35, dt (16.8, 4.8) 2.68, ddd (16.8, 13.1, 4.8)	2.37, dt (16.7, 4.9) 2.69, ddd (16.7, 13.2, 4.9)	2.35, dt (17.1, 4.7) 2.61, m^c^	1.72, m 1.85, m^c^
7*α* 7*β*	1.66, ddd (13.5, 7.4, 2.8) 1.51, overlapped	2.02, m 1.90, m	2.03, m 1.92, m	1.82, overlapped 1.89, td (13.1, 4.7)	1.84, m
9*α* 9*β*	1.45, ddd (14.0, 4.4, 3.3) 2.21, td (14.0, 3.3)	1.59, dt (14.4, 3.1) 2.47, td (14.4, 3.1)	1.62, ddd (14.3, 4.4, 3.1) 2.48, td (14.0, 3.1)	1.80, overlapped 1.60, td (13.9, 3.5)	3.89, br.d (11.6)
10*α*	1.54, td (14.0, 3.3)	1.73, td (13.6, 3.1)	1.75, td (13.8, 3.1)	2.14, m	2.16, m
10*β*	1.84, ddd (14.0, 4.4, 3.3)	1.94, overlapped	1.95, overlapped	1.46, m^c^	1.74, overlapped^c^
12				1.97, d (2.7)	1.57, overlapped
13					2.47, dd (18.1, 7.6) 2.14, m
15	1.72, s	1.67, s	4.26, dd (11.9, 5.9) 4.11, dd (11.9, 5.9)	1.84, br.s	5.22, d (1.4) 5.11, d (1.4)
16	0.86, s	1.07, s	1.10, s	1.11, s	0.66, s
17	1.12, s	1.12, s	1.15, s	1.11, s	1.16, s
18	2.52, h (7.0)	2.54, h (7.1)	2.55, h (7.0)	2.07, m	1.82, m
19^d^	1.26, d (7.0)	1.30, d (7.1)	1.30, d (7.0)	1.14, d (6.9)	0.93, d (6.7)
20^d^	1.29, d (7.0)	1.28, d (7.1)	1.28, d (7.0)	0.84, d (6.9)	1.07, d (6.7)
5-OH	3.98, d (6.7)				
13-OH	7.71, br. s	7.91, br. s	7.96, br. s		
14-OH				7.84, br. s	
15-OH			3.62, t (5.9)		

^a^In Me_2_CO-*d*_6_

^b^In CDCl_3_

^c^Could not be assigned to *α/β*, signals overlapping

^d^Signals were exchangeable

**Table 2:** ^13^C NMR data of guanacastepenes P–T (**1**–**5**)

No.	**1** ^a^	**2** ^b^	**3** ^a^	**4** ^a^	**5** ^c^
1	141.6, qC	142.3, qC	143.1, qC	148.8, qC	147.5, qC
2	130.4, CH	127.9, CH	127.3, CH	30.3, CH_2_	134.2, CH
3	136.4, qC	157.2, qC	160.4, qC	160.5, qC	40.5, CH
4	139.1, qC	132.7, qC	136.3, qC	131.8, qC	148.6, qC
5	70.3, CH	197.5, qC	198.0, qC	197.5, qC	73.9, CH
6	29.7, CH_2_	34.1, CH_2_	34.3, CH_2_	34.2, CH_2_	30.0, CH_2_
7	38.0, CH_2_	38.9, CH_2_	38.9, CH_2_	39.9, CH_2_	31.5, CH_2_
8	36.7, qC	37.5, qC	37.8, qC	40.2, qC	44.3, qC
9	38.5, CH_2_	35.5, CH_2_	35.5, CH_2_	41.3, CH_2_	77.2, CH
10	30.9, CH_2_	30.7, CH_2_	30.8, CH_2_	36.1, CH_2_	43.7, CH_2_
11	44.7, qC	44.6, qC	44.7, qC	45.0, qC	44.0, qC
12	154.9, qC	155.7, qC	155.9, qC	62.0, CH	51.8, CH
13	151.0, qC	151.2, qC	151.3, qC	202.9, qC	41.3, CH_2_
14	189.1, qC	188.5, qC	188.5, qC	148.1, qC	205.2, qC
15	17.0, CH_3_	12.2, CH_3_	56.4, CH_2_	11.7, CH_3_	111.9, CH_2_
16	26.5, CH_3_	26.1, CH_3_	25.9, CH_3_	24.3, CH_3_	10.5, CH_3_
17	20.7, CH_3_	20.6, CH_3_	20.8, CH_3_	17.8, CH_3_	17.2, CH_3_
18	26.5, CH	26.5, CH	26.6, CH	28.1, CH	28.7, CH
19^d^	20.8, CH_3_	20.7, CH_3_	20.7, CH_3_	23.7, CH_3_	22.6, CH_3_
20^d^	20.8, CH_3_	20.7, CH_3_	20.7, CH_3_	19.2, CH_3_	24.3, CH_3_

^a^Recorded at 150 MHz in Me_2_CO-*d*_6_

^b^Recorded at 100 MHz in Me_2_CO-*d*_6_

^c^Recorded at 150 MHz in CDCl_3_

^d^Signals were exchangeable

Guanacastepene S (**4**) had the same molecular formula of C_20_H_28_O_3_ (HRESIMS ([M+Na]^+^ at *m*/*z* 339.1927) as **1** and was obtained as a colorless oil. Its ^13^C NMR spectrum (Table 2) displayed 20 carbon signals corresponding to five methyls (three singlets and two doublets), five methylenes, two methines and eight quaternary carbons (four olefinic and two carbonyl carbons), consistent with a similar structure of compound **2**. Significant differences were one less double bond and absence of an olefinic hydrogen in **4**. The H-18 correlation with a methine proton at *δ*_H_ 1.97 (1H, d, *J* = 2.7 Hz, H-12) in the ^1^H–^1^H COSY spectrum, and the key HMBC correlations of the OH signal at *δ*_H_ 7.84 (1H, br. s, OH-14) to a keto carbonyl carbon *δ*_C_ 202.9 (s, C-13) and the double bond signal at *δ*_C_ 148.1 (s, C-14) indicated a different enolization of the 13,14-diketone system (to a 13-keto-14-enol) in **4**. Further analysis of 2D NMR spectroscopic data suggested that the other parts were the same to those of **2**, except the olefinic carbon at C-2 in **2** being replaced by a methylene moiety in **4**, as established by the HMBC correlations of *δ*_H_ 3.50 and 3.60 (each 1H, d, *J* = 19.0 Hz, H-2) to C-1. On the basis of the ROESY experiment, H-12 was elucidated to be *α* oriented by correlations of H-17 with H-18 and H-19. Thus compound **4** was assigned as guanacastepene S.

Guanacastepene T (**5**) possessed the molecular formula C_20_H_30_O_3_, as determined by its HRESIMS at *m*/*z* 341.2091 ([M+Na]^+^), indicating six degrees of unsaturation. The ^13^C NMR spectroscopic data (Table 2) together with analysis of its HSQC spectrum indicated 20 carbons, including four methyls, five methylenes (one olefinic), six methines (two oxygenated and one olefinic), and five quaternary carbons (two olefinic and one carbonyl carbons). Comprehensive analysis of the ^1^H–^1^H COSY and HMBC spectra (Fig. 2), suggested that **5** shared the same guanacastepene skeleton with **1**, except for differences in the number and location of functional groups. The ^1^H–^1^H COSY correlations of H-19/H-18/H-12/H-13, combined with HMBC correlations of H-13 with C-14, C-12 and C-1, and H-2 with C-1 and C-14 suggested the presence of a single 2(1)-en-14-one system in **5**. Besides, the trisubstituted double bond between C-3 and C-4 in **1** was changed into a terminal double bond between C-4 and C-15 in **5**, consistent with the ^1^H NMR chemical shifts of *δ*_H_ 5.11 and 5.22 (each 1H, br. s, H-15), which was also supported by the HMBC correlations of H-15 to C-3 and C-4, and H-3 to C-2 and C-4. The ^1^H–^1^H COSY data, as well as HMBC correlations of H-5 to C-3 and C-15 also gave the information that C-5 was a methine substituted by a hydroxy group. Likewise, C-9 was confirmed as a hydroxy substituted methine based on the ^1^H–^1^H COSY and HMBC correlations as shown in Fig. 2. Based on the proposed biogenetic orientation of Me-16 and Me-17, the configurations of H-3 and HO-9 were determined to be *β* and *α* oriented, respectively, owing to the strong correlation signals of H-3 with H-17 and H-9, and H-9 with H-17 in the ROESY spectrum. In addition, the stereoconfiguration of H-5 was determined to be *α* oriented, as indicated by the observation of the ROESY correlations the same to those of compound **1**. Consequently, compound **5** was elucidated as guanacastepene T.

Compounds **1**–**5** were evaluated for their cytotoxicity against five human cancer cell lines. None was found to possess significant activity with IC_50_ values less than 40 μM. In addition, they were evaluated for anti-HSV (herpes simplex viruses) activity. However, none exhibited activity. Furthermore, compounds **1**, **3** and **5** were tested for inhibitory activities against one isozyme of 11*β*-HSD1. Of these, only compound **3** exhibited inhibitory activity against both human and mouse isozymes of 11*β*-HSD1 with IC_50_ values of 6.2 and 13.9 μM, while glycyrrhizinic acid (positive control) had IC_50_ values of 4.2 and 6.5 nM, respectively.

Five new guanacastane-type diterpenes, guanacastepenes P–T, each containing a 5/7/6 ring system, were isolated from cultures of fungus *P. candolleana*. Until now, totally 33 guanacastane-type diterpenes have been obtained from kinds of fungi, and some of them showed antibiotic and antitumor activities. Our present research enriched the structure diversity of the guanacastane family and this is also the first report to show that guanacastane-type diterpenes possessed inhibitory activity against human 11β-HSD1.

## Experimental

### General Experimental Procedures

Optical rotations (OR) were recorded on a JASCO P-1020 digital polarimeter, while the UV and IR spectra were obtained on a Shimadzu UV2401PC and a Bruker Tensor 27 FT-IR (KBr pellets) spectrometers. NMR spectra were acquired on Bruker AM-400, DRX-500 and Avance III 600 MHz spectrometers with tetramethylsilane (TMS) used as an internal standard at room temperature. High-resolution (HR) ESI-MS were recorded on an API QSTAR Pulsar spectrometer. Silica gel (200–300 mesh, Qingdao Marine Chemical Ltd., People’s Republic of China) and Sephadex LH-20 (Amersham Biosciences, Sweden) were used for open column chromatography (CC). Preparative HPLC was performed on an Agilent 1100 liquid chromatography system equipped with a Zorbax SB-C18 column (21.2 mm × 150 mm). Fractions were monitored by TLC. Spots were visualized by heating silica gel plates immersed in vanillin–H_2_SO_4_ in EtOH.

### Fungal Material and Cultivation Conditions

The fungus *P. candolleana* (Pers.) Fr. was collected at Kunming Institute of Botany in Yunnan Province, People’s Republic of China, in 2003. The fungus was identified by Prof. Zhu-Liang Yang at the Kunming Institute of Botany. A specimen (No. KIB20030828) was deposited at Kunming Institute of Botany, Chinese Academy of Sciences.

Basidiomata small to medium-sized. Pileus 3–7 cm in diam, campaniform then flattened, with moist umbonate center; surface glabrous, light honey-yellow to brown, yellow-brown at the apex, fading to grayish when dry; white universal veil gradually fall off with age. Context white, relatively thin. Lamellae adnate, narrow and length unequal, crowded, dirty white, grayish to pallid purple-brown; edge dirty white, coarse. Stipe slender and fistulous, cylindrical, 3–8 × 0.2–0.7 cm, white, weak and fragile, surface densely floccose-fibrillose or reticulate. Spores print dark purple-brown; spores smooth, ellipsoid, 6.5–9.0 × 3.5–5.0 μm, germ pore was visible. Pleurocystidia smooth and thin-walled, hyaline in KOH, more or less lageniform and apex rounded, 34–50 × 8–16 μm.

The culture medium consisted of glucose (5 %), peptone from porcine meat (0.15 %), yeast powder (0.5 %), KH_2_PO_4_ (0.5 %) and MgSO_4_. Fermentation was carried out on a shaker at 160 RPM for 25 days.

### Extraction and Isolation

The culture broth (21 L) was filtered, and the filtrate was extracted with ethyl acetate (20 L × 3), while the mycelium was extracted three times with CHCl_3_–MeOH (1:1). The EtOAc layer together with the mycelium extract was concentrated under reduced pressure to give a crude extract (10 g), and the latter was applied to a silica gel column eluted with a gradient of CHCl_3_/MeOH (1:0→0:1) to obtain five fractions (1–5). Fraction 3 was subjected to Sephadex LH-20 (CHCl_3_/MeOH 1:1) to give two subfractions (3a–3b). Fraction 3a was separated by semipreparative HPLC (MeCN/H_2_O, 1:9→2:8, 30 min) to give three mixtures, then purified separately by Sephadex LH-20 CC (acetone) to give **2** (2.1 mg), **4** (2.0 mg) and **5** (4.8 mg), respectively. In the same way, subfraction 3b was purified by semipreparative HPLC (MeCN/H_2_O, 1:9→2:8, 40 min) to yield **1** (3.1 mg) and **3** (3.3 mg).

### Guanacastepene P (**1**)

Light yellow oil; $[\alpha ]_{\text{D}}^{ 1 6}$ − 507.0 (*c* 0.70, MeOH); IR (KBr) *ν*_max_ 3444, 2925, 1632 cm^−1^; UV (MeOH) *λ*_max_ (log *ε*) 307 (4.16), 206 (4.16); For ^1^H (400 MHz) and ^13^C NMR (150 MHz) spectroscopic data (Me_2_CO-*d*_6_), see Tables 1 and 2; positive ion HRESIMS *m*/*z* 339.1930 (calcd for C_20_H_28_O_3_Na [M+Na]^+^, 339.1936).

### Guanacastepene Q (**2**)

Light yellow oil; $[\alpha ]_{\text{D}}^{ 1 6}$ − 318.3 (*c* 1.6, MeOH); IR (KBr) *ν*_max_ 3433, 2963, 2925, 1669, 1627, 1409, 1152 cm^−1^; UV (MeOH) *λ*_max_ (log *ε*) 289 (4.03), 236 (3.87), 203 (3.91); For ^1^H (400 MHz) and ^13^C NMR (100 MHz) spectroscopic data (Me_2_CO-*d*_6_), see Tables 1 and 2; positive ion HRESIMS *m*/*z* 315.1962 (calcd for C_20_H_27_O_3_ [M+H]^+^, 315.1960).

### Guanacastepene R (**3**)

Light yellow oil; $[\alpha ]_{\text{D}}^{ 1 6}$ − 303.7 (*c* 1.9, MeOH); IR (KBr) *ν*_max_ 3433, 2924, 1630, 1104 cm^−1^; UV (MeOH) *λ*_max_ (log *ε*) 285 (3.99), 232 (3.82), 203 (3.93); For ^1^H (400 MHz) and ^13^C NMR (150 MHz) spectroscopic data (Me_2_CO-*d*_6_), see Tables 1 and 2; positive ion HRESIMS *m*/*z* 353.1737 (calcd for C_20_H_26_O_4_ [M+H]^+^, 353.1728).

### Guanacastepene S (**4**)

Colorless oil; $[\alpha ]_{\text{D}}^{ 2 1}$ − 167.5 (*c* 1.9, MeOH); IR (KBr) *ν*_max_ 3431, 2924, 2346, 1630 cm^−1^; UV (MeOH) *λ*_max_ (log *ε*) 285 (3.82), 201 (3.86); For ^1^H (400 MHz) and ^13^C NMR (150 MHz) spectroscopic data (Me_2_CO-*d*_6_), see Tables 1 and 2; positive ion HRESIMS *m*/*z* 339.1927 (calcd for C_20_H_28_O_3_ [M+Na]^+^, 339.1927).

### Guanacastepene T (**5**)

Colorless oil; $[\alpha ]_{\text{D}}^{ 2 1}$ + 0.75 (*c* 0.8, MeOH); IR (KBr) *ν*_max_ 3440, 2923, 1631 cm^−1^; UV (MeOH) *λ*_max_ (log *ε*) 252 (3.71), 201(3.52); For ^1^H (400 MHz) and ^13^C NMR (150 MHz) spectroscopic data (CDCl_3_), see Tables 1 and 2; positive ion HRESIMS *m*/*z* 341.2091 (calcd for C_20_H_30_O_3_ [M+Na]^+^, 341.2092).

### Cytotoxicity Assay

Human myeloid leukemia HL-60, hepatocellular carcinoma SMMC-7721, lung cancer A-549 cells, breast cancer MCF-7 and colon cancer SW480 cell lines were used in the cytotoxic assay. All cell lines were cultured in RPMI-1640 or DMEM medium (Hyclone, USA), supplemented with 10 % fetal bovine serum (Hyclone, USA) in 5 % CO_2_ at 37 °C. The cytotoxicity assay was performed according to the MTT [3-(4,5-dimethylthiazol-2-yl)-2,5-diphenyl tetrazolium bromide[ method in 96-well microplates [21]. Briefly, adherent cells (100 µL) were seeded into each well of 96-well cell culture plates and allowed to adhere for 12 h before drug addition, while suspended cells were seeded just before drug addition with initial density of 1 × 10^5^ cells/mL. Each tumor cell line was exposed to the test compound dissolved in DMSO at concentrations of 0.0625, 0.32, 1.6, 8, and 40 μM in triplicates for 48 h, with *cis*platin (Sigma, USA) as a positive control. After compound treatment, cell viability was detected and a cell growth curve was graphed. IC_50_ values were calculated by Reed and Muench’s method [22].

### Antiviral Assay

Confluent Vero cells in a 96-well tissue plate were inoculated in triplicate with virus suspension (50 μL) [HSV/Blue, at multiplicity of infection (MOI) 1] and culture medium (50 μL) containing testing compounds at different concentrations. Cells were lysed with 1 % Nonidet P-40 in DMEM at 24 h postinfection. Lysates from each well were mixed with chlorophenol red-*β*-d-galactopyranoside [CPRG; Boehringer, Ingelheim, Germany), and *β*-galactosidase (*β*-Gal)] activity was measured by taking absorbance readings at 570 nm every 2 min for a total of 25 readings. The slope of the line was used to quantify *β*-Gal activity as milli-optical density units/min (mOD/min). The 50 % inhibitory concentration (IC_50_) was defined as the concentration of the antiviral drug that reduced the mOD/min values by 50 % relative to the virus control. Inhibitory concentrations were calculated using the probit regression method [23].

### Inhibitory Activities Against 11β-HSD1 Assay

Inhibitory activities of the compounds on human or mouse 11*β*-HSD1 were determined using scintillation proximity assay (SPA). Microsomes containing 11*β*-HSD1 were used according to our previous studies [24, 25]. Full-length cDNAs of human or murine 11*β*-HSD1 were isolated from cDNA libraries provided by the NIH Mammalian Gene Collection. The cDNAs were cloned into pcDNA3 expression vectors. HEK-293 cells were transfected with the pcDNA3-derived expression plasmid and selected by cultivation in the presence of G418 (700 μg/mL). The microsomal fraction overexpressing 11*β*-HSD1 was prepared from the HEK-293 cells, which were stable transfected with 11*β*-HSD1. The fraction was then used as the enzyme source for SPA. Microsomes containing human or mouse 11*β*-HSD1 were incubated with NADPH and [^3^H] cortisone. The product, [^3^H] cortisol, was specifically captured by a monoclonal antibody coupled to protein A-coated SPA beads. All tests were done in twice with glycyrrhizinic acid as a positive control. IC_50_ (X ± SD, *n* = 2) values were calculated by using Prism Version 4 (GraphPad Software, San Diego, CA, USA).

## Electronic supplementary material

Below is the link to the electronic supplementary material.

Supplementary material 1 (DOC 4075 kb)
