# Supplementary material for: Five New Guanacastane-Type Diterpenes from Cultures of the Fungus Psathyrella candolleana
Source: Nat Prod Bioprospect. 2014 May 8;4(3):149–55. doi: 10.1007/s13659-014-0020-8 (PMC4050309; doi:10.1007/s13659-014-0020-8)

Five New Guanacastane-Type Diterpenes from Cultures of the Fungus *Psathyrella candolleana*

Xia Yin, ^a,b^ Tao Feng,^a^ Zheng-Hui Li,^a^ Ying Leng,^c^ and Ji-Kai Liu ^a^, *^*^*

^a^ State Key Laboratory of Phytochemistry and Plant Resources in West China, Kunming Institute of Botany, Chinese Academy of Sciences, Kunming 650201, People’s Republic of China,

^b^ University of Chinese Academy of Sciences, Beijing 100049, People’s Republic of China,

^c^ Shanghai Institute of Materia Medica, Chinese Academy of Sciences, Shanghai 201203, People’s Republic of China

*E-mail addresses*: jkliu@mail.kib.ac.cn

**Supporting Information**

Figure 1S-7S. NMR and MS spectra of Guanacastepene P (**1**)

Figure 8S-14S. NMR and MS spectra of Guanacastepene Q (**2**)

Figure 15S-21S. NMR and MS spectra of Guanacastepene R (**3**)

Figure 22S-28S. NMR and MS spectra of Guanacastepene S (**4**)

Figure 29S-35S. NMR and MS spectra of Guanacastepene T (**5**)

Figure 1S. ^1^H NMR of Guanacastepene P (**1**)


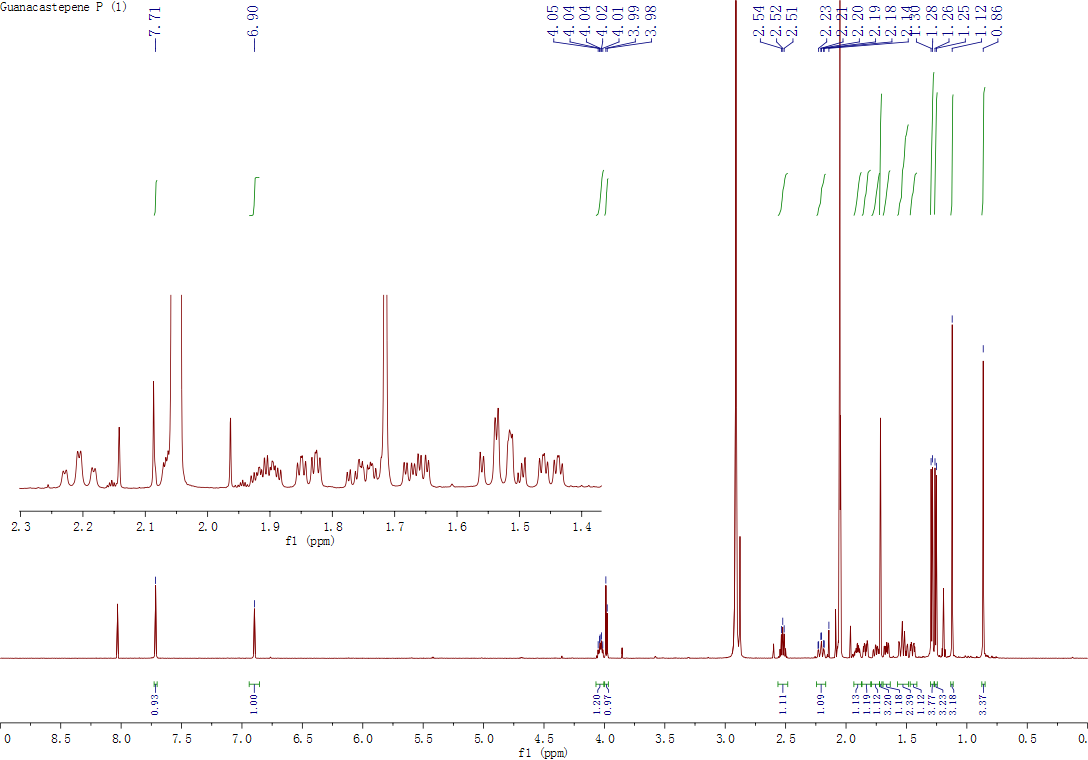


Figure 2S. ^13^C NMR and DEPT of Guanacastepene P (**1**)


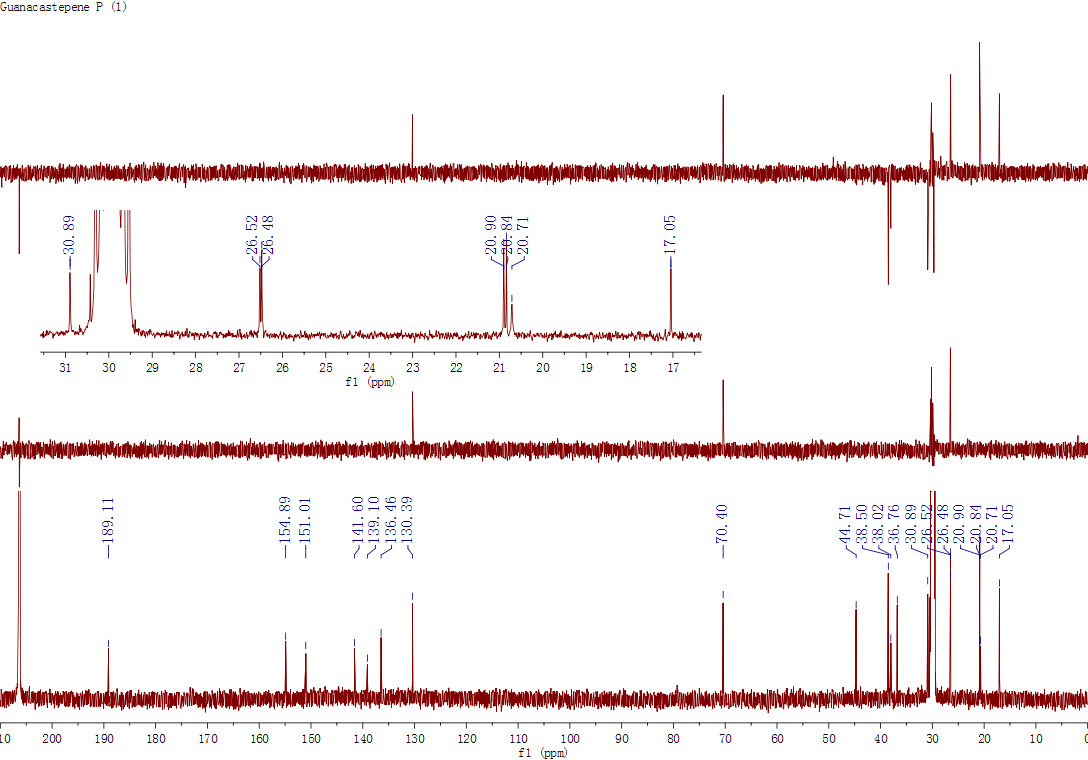


Figure 3S. HSQC of Guanacastepene P (**1**)


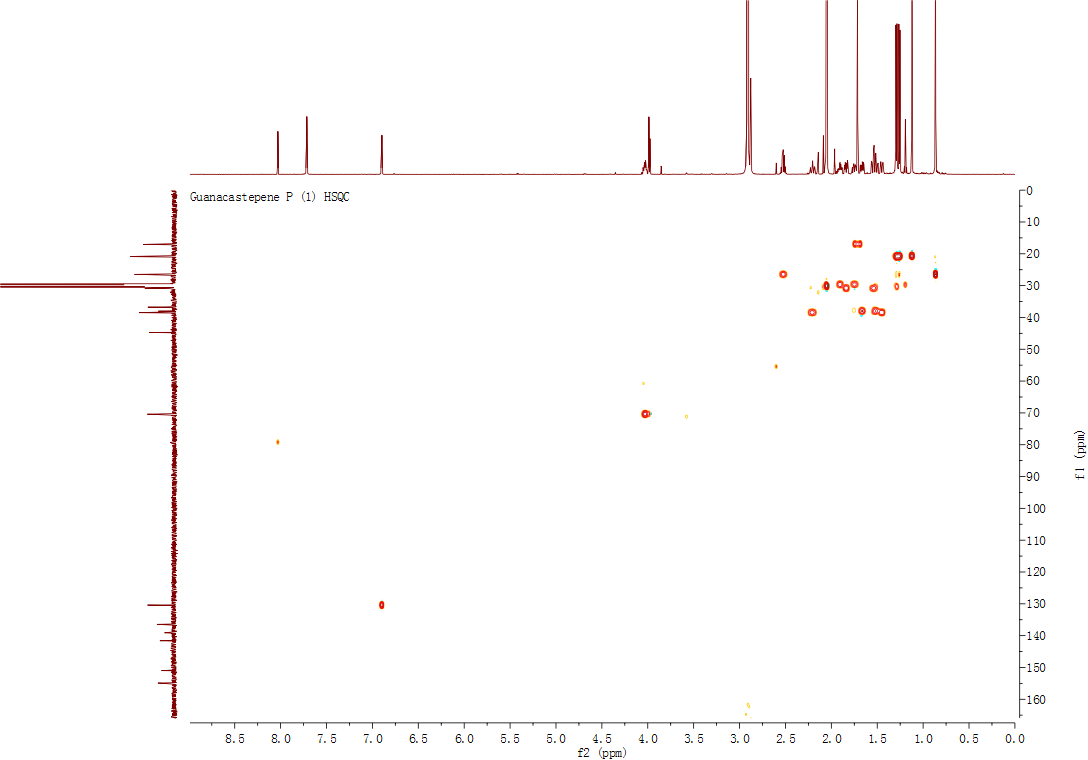


Figure 4S. HMBC of Guanacastepene P (**1**)


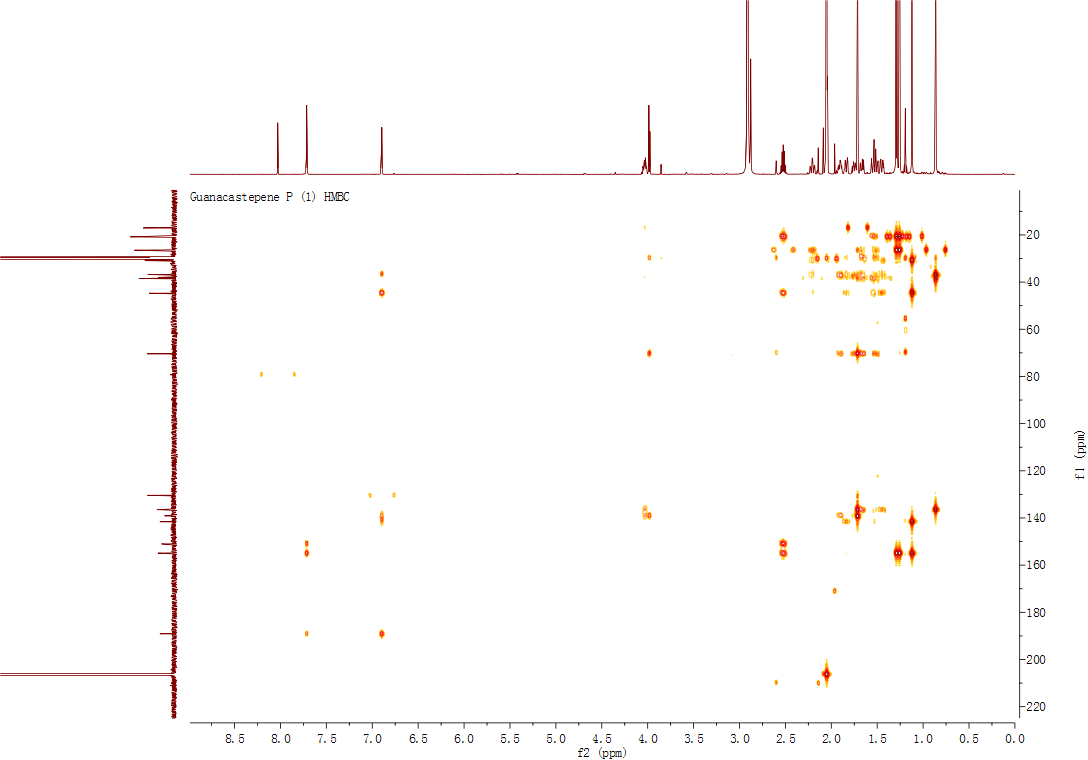


Figure 4΄S. enlarged HMBC of Guanacastepene P (**1**)


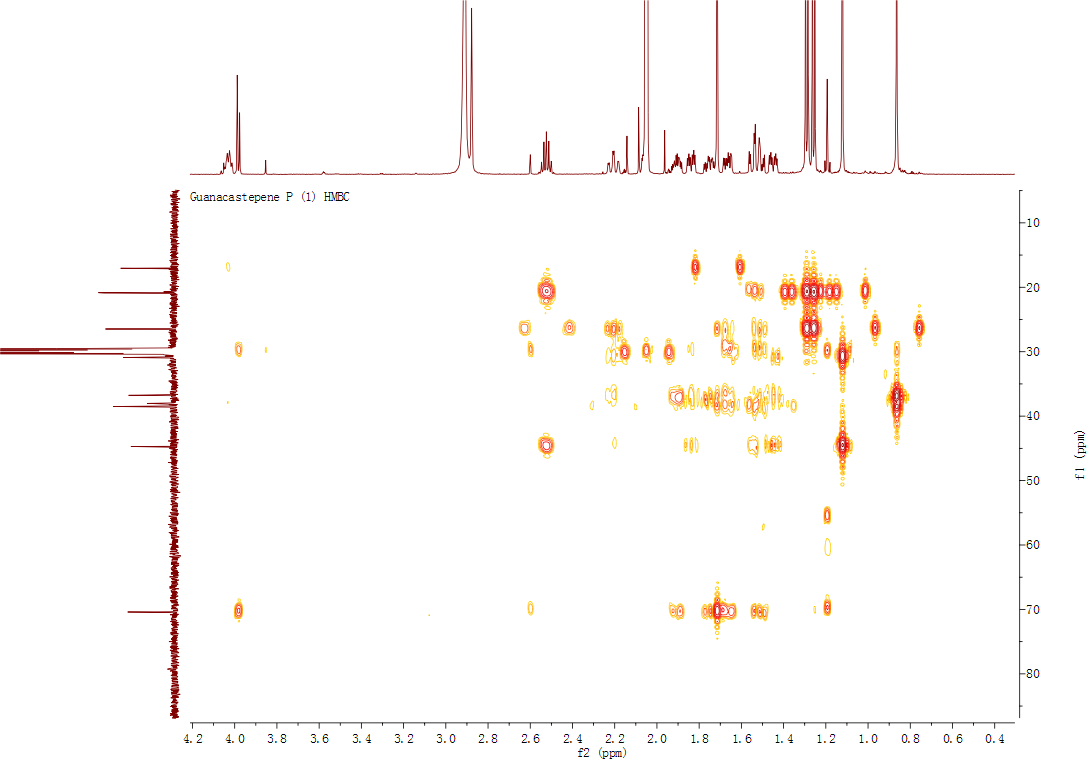


Figure 5S. ^1^H-^1^H COSY of Guanacastepene P (**1**)


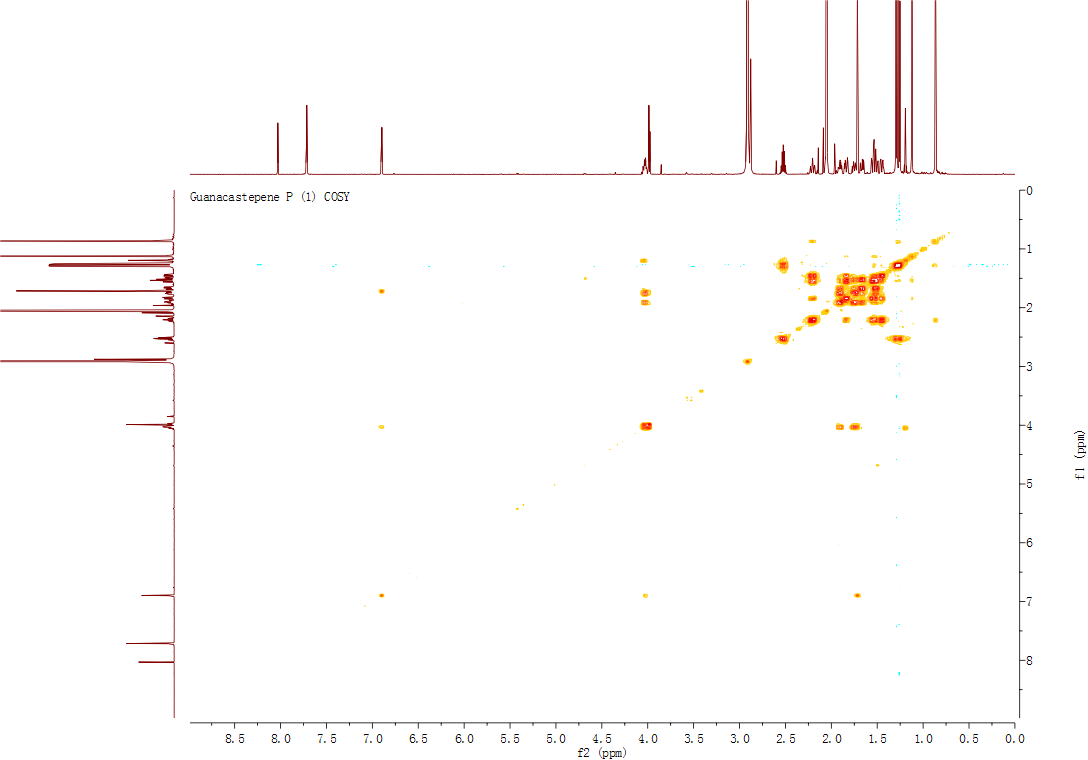


Figure 5΄S. enlarged ^1^H-^1^H COSY of Guanacastepene P (**1**)


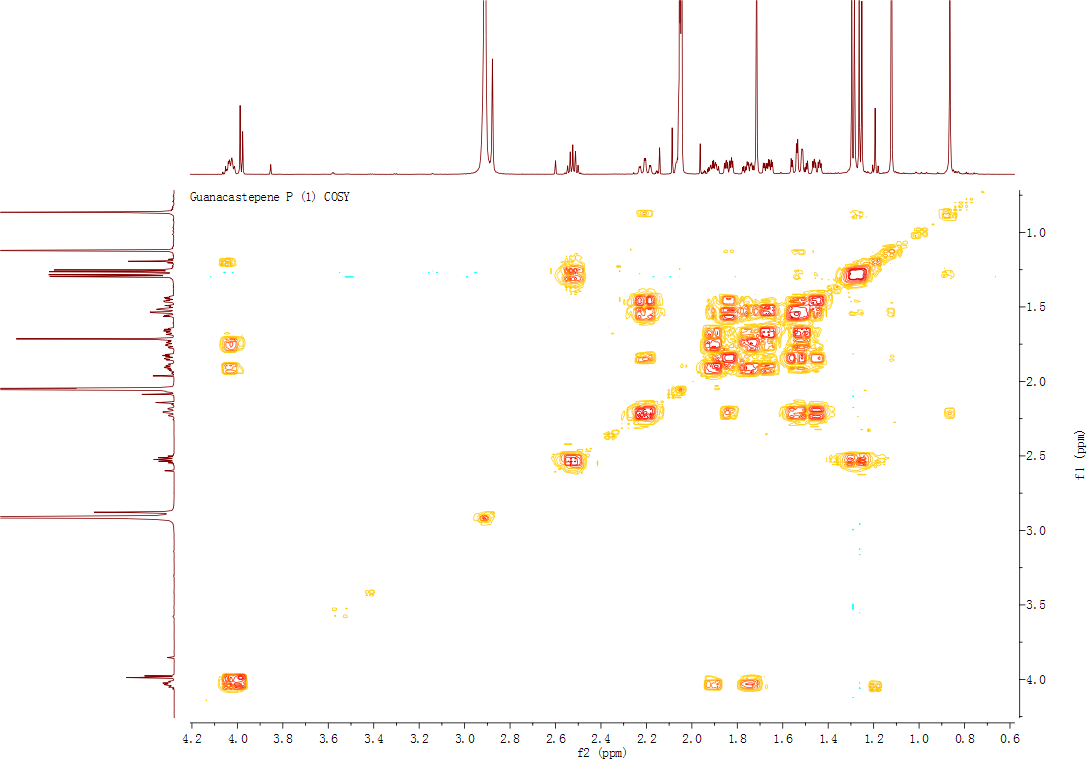


Figure 6S. ROESY of Guanacastepene P (**1**)


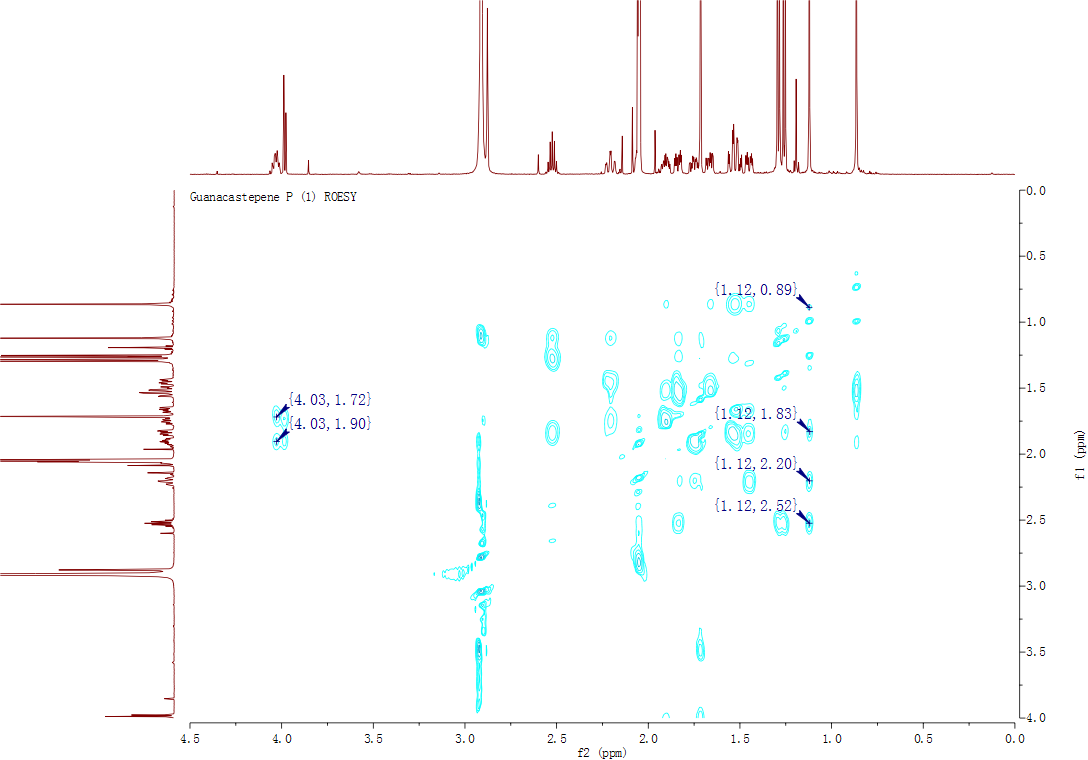


Figure 7S. HRESIMS of Guanacastepene P (**1**)


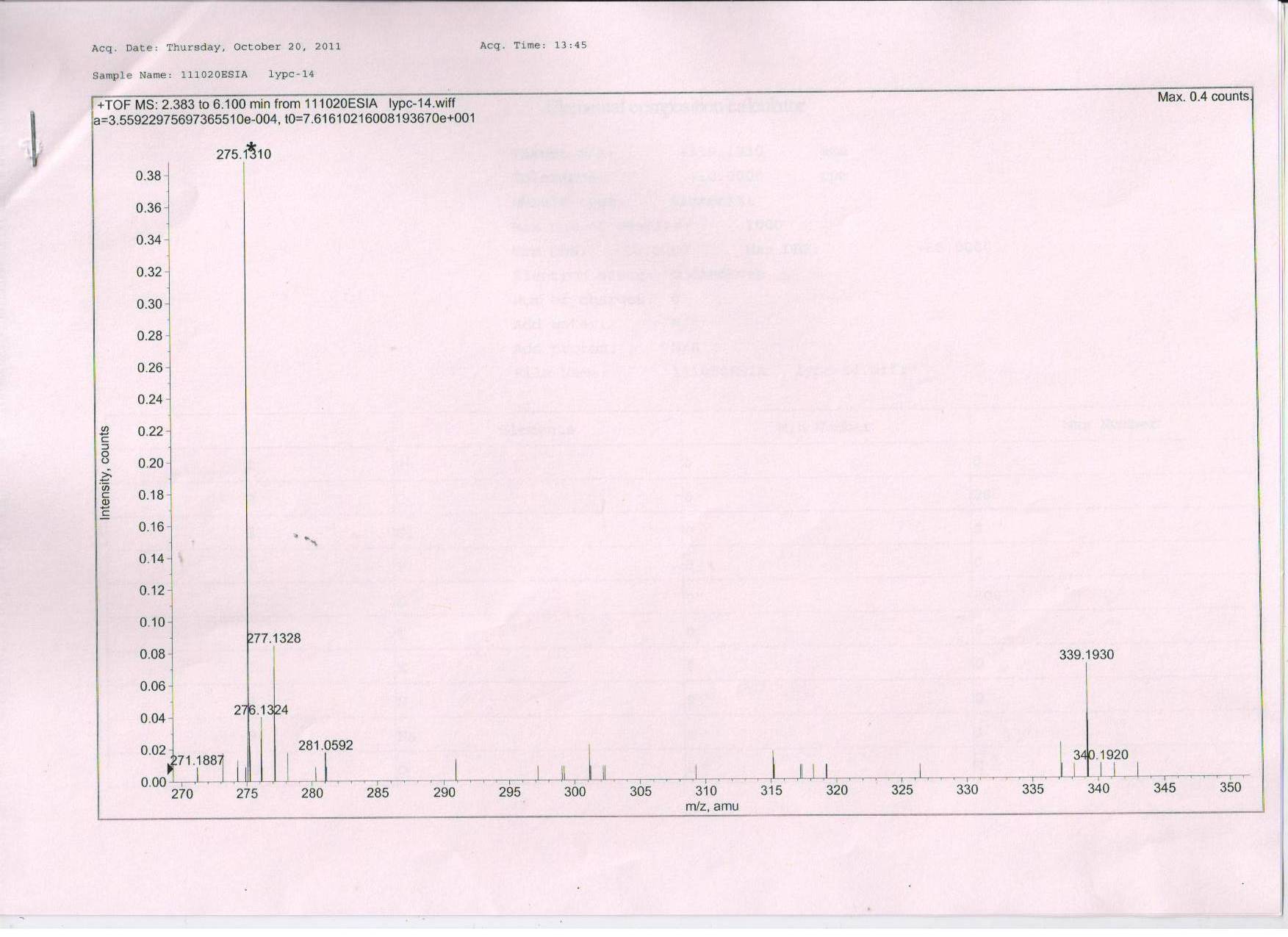


Figure 8S. ^1^H NMR of Guanacastepene Q (**2**)


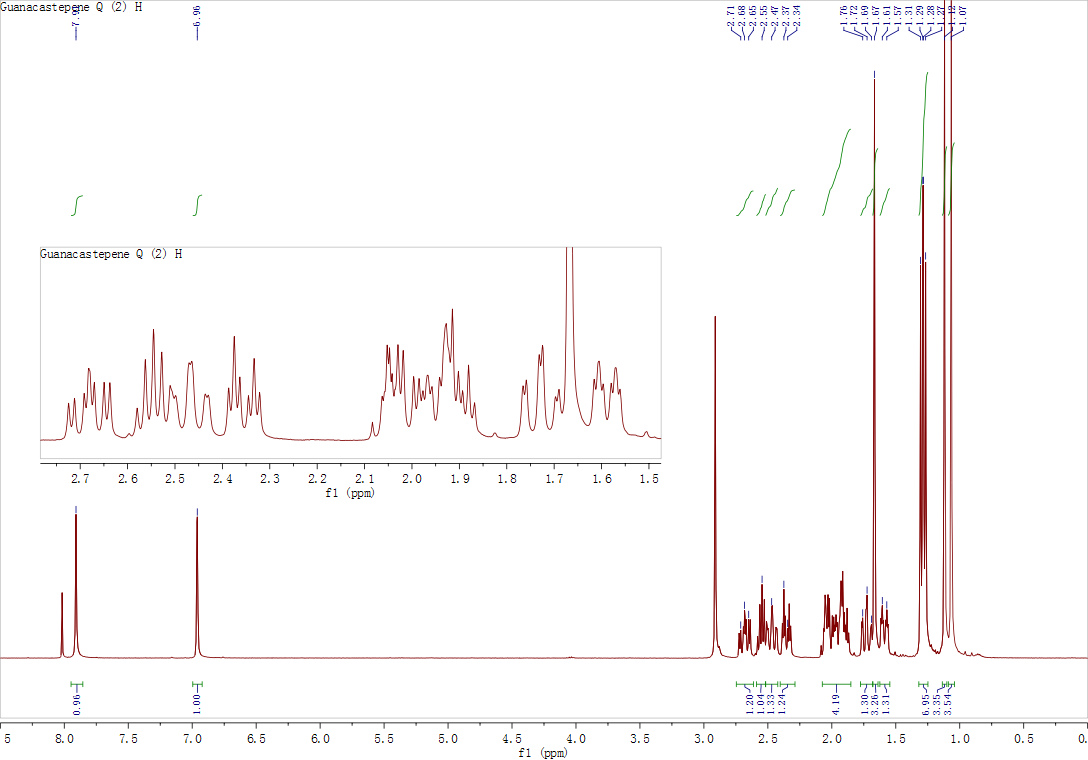


Figure 9S. ^13^C NMR and DEPT of Guanacastepene Q (**2**)


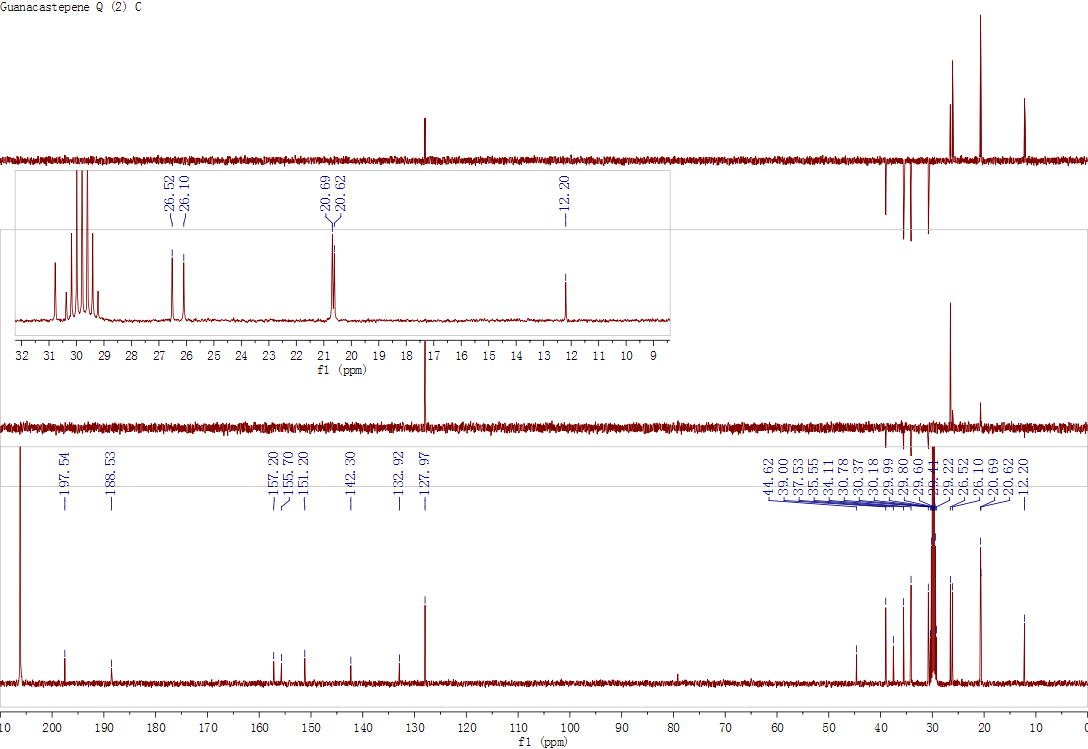


Figure 10S. HSQC of Guanacastepene Q (**2**)


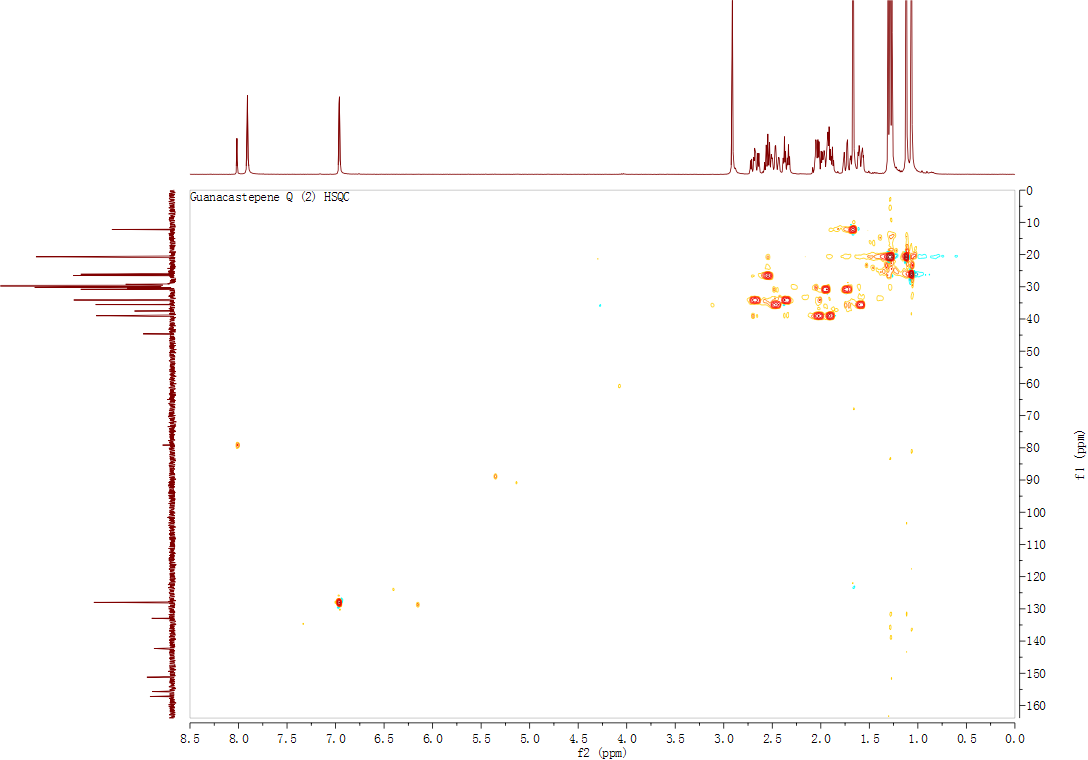


Figure 11S. HMBC of Guanacastepene Q (**2**)


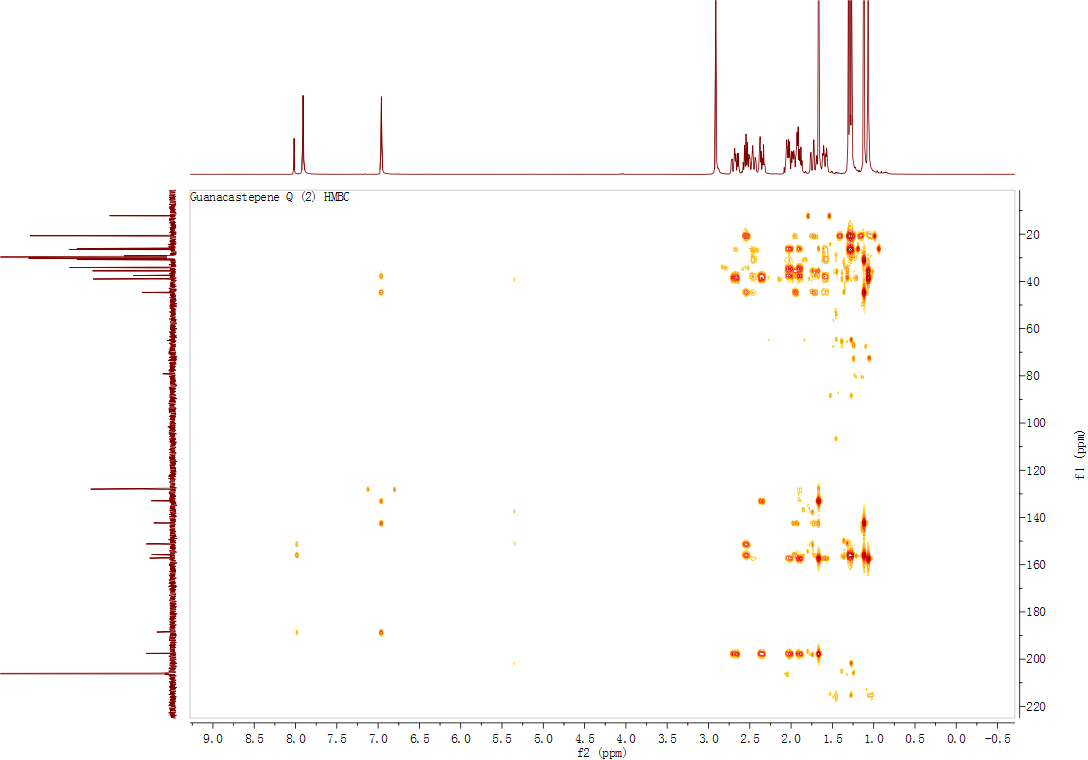


Figure 11΄S. enlarged HMBC of Guanacastepene Q (**2**)


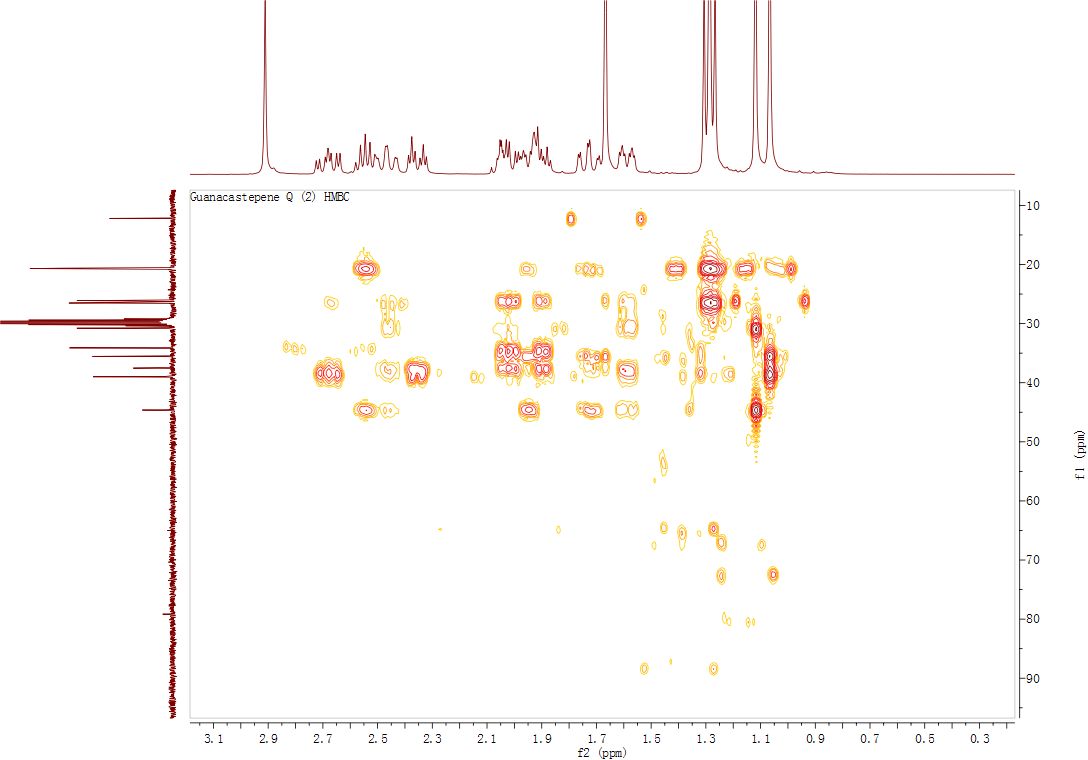


Figure 12S. ^1^H-^1^H COSY of Guanacastepene Q (**2**)


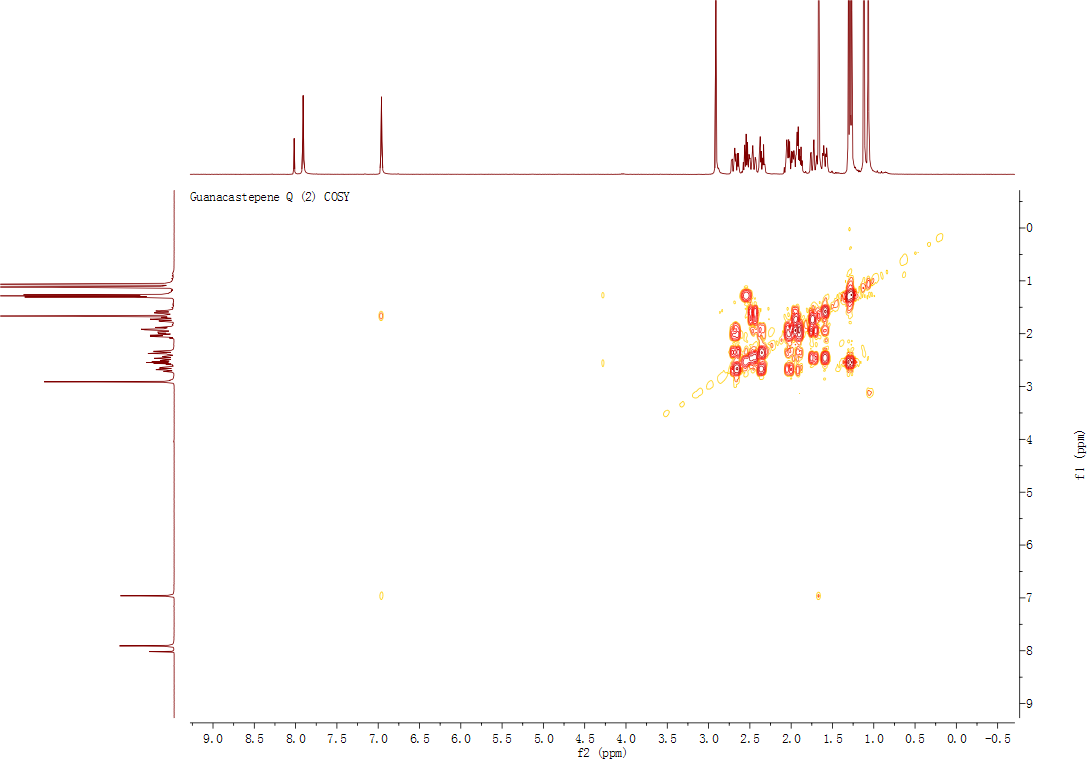


Figure 12΄S. enlarged ^1^H-^1^H COSY of Guanacastepene Q (**2**)


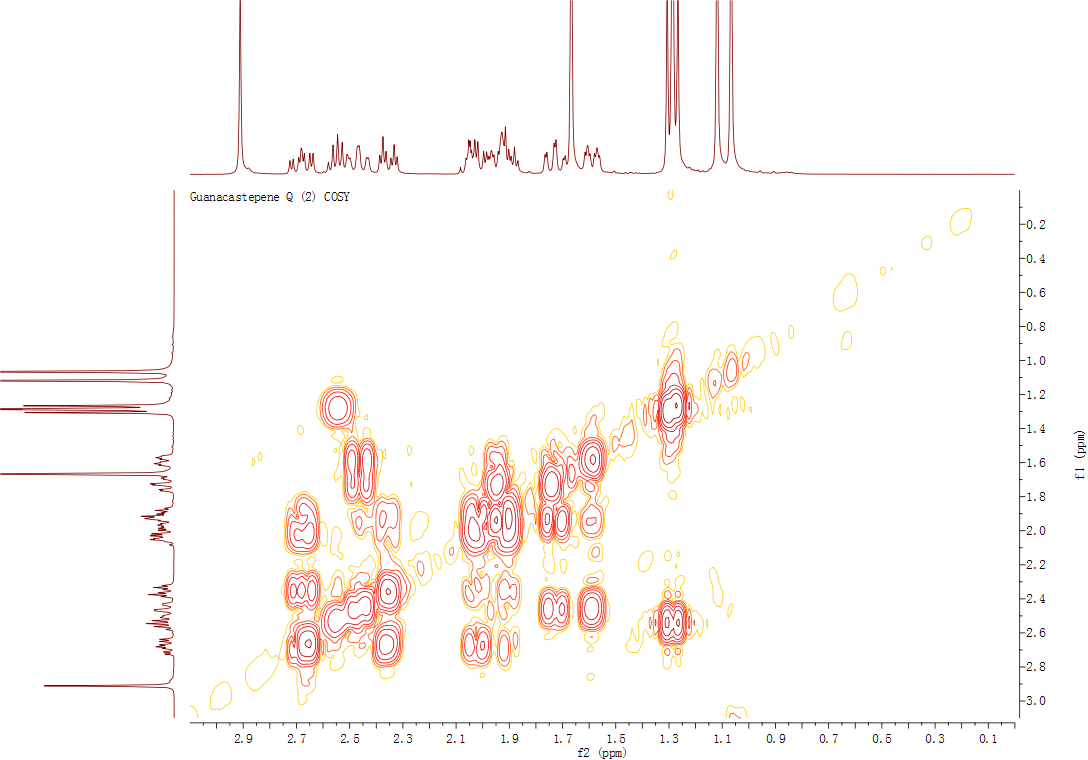


Figure 13S. ROESY of Guanacastepene Q (**2**)


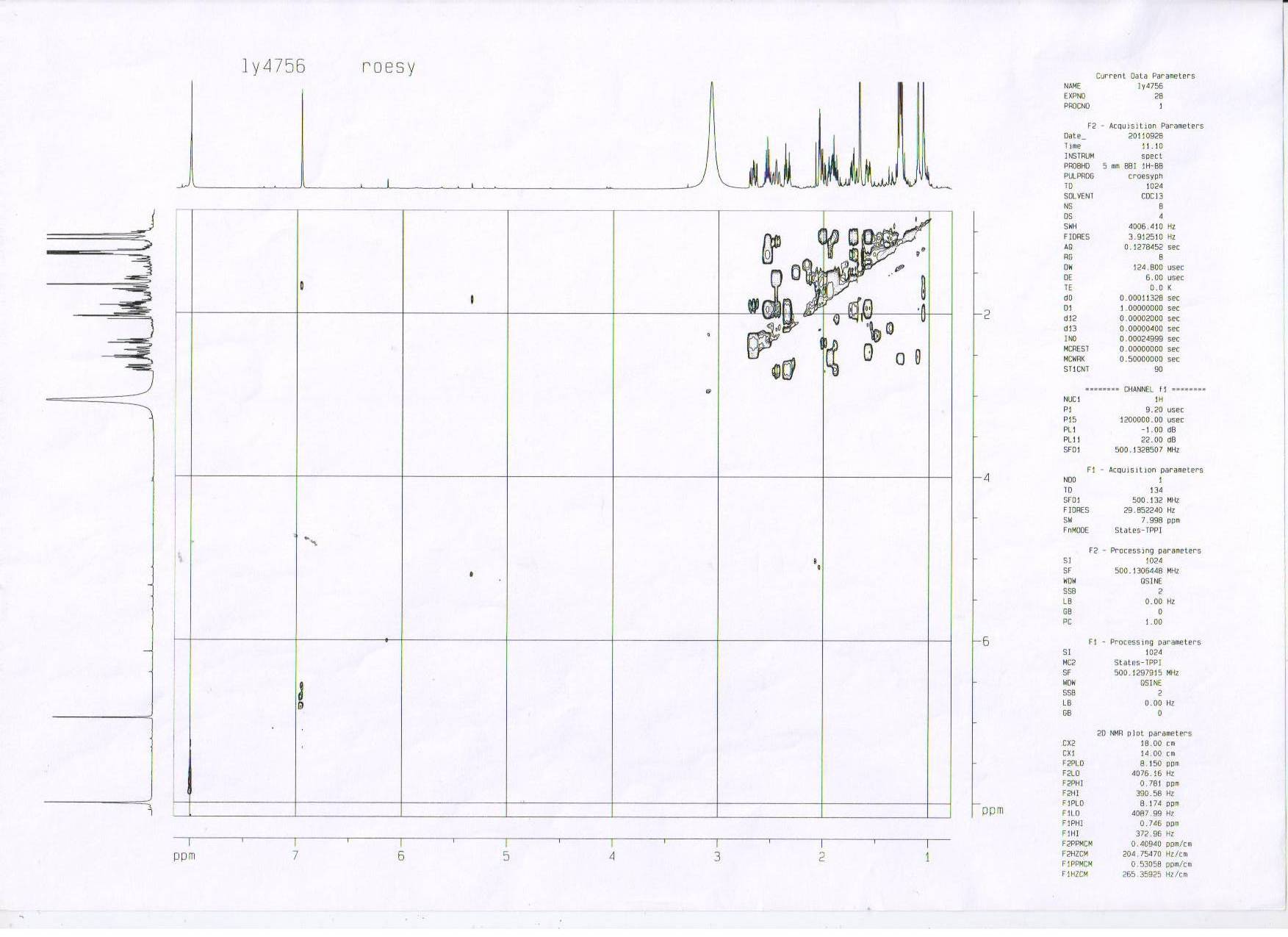


Figure 14S. HRESIMS of Guanacastepene Q (**2**)


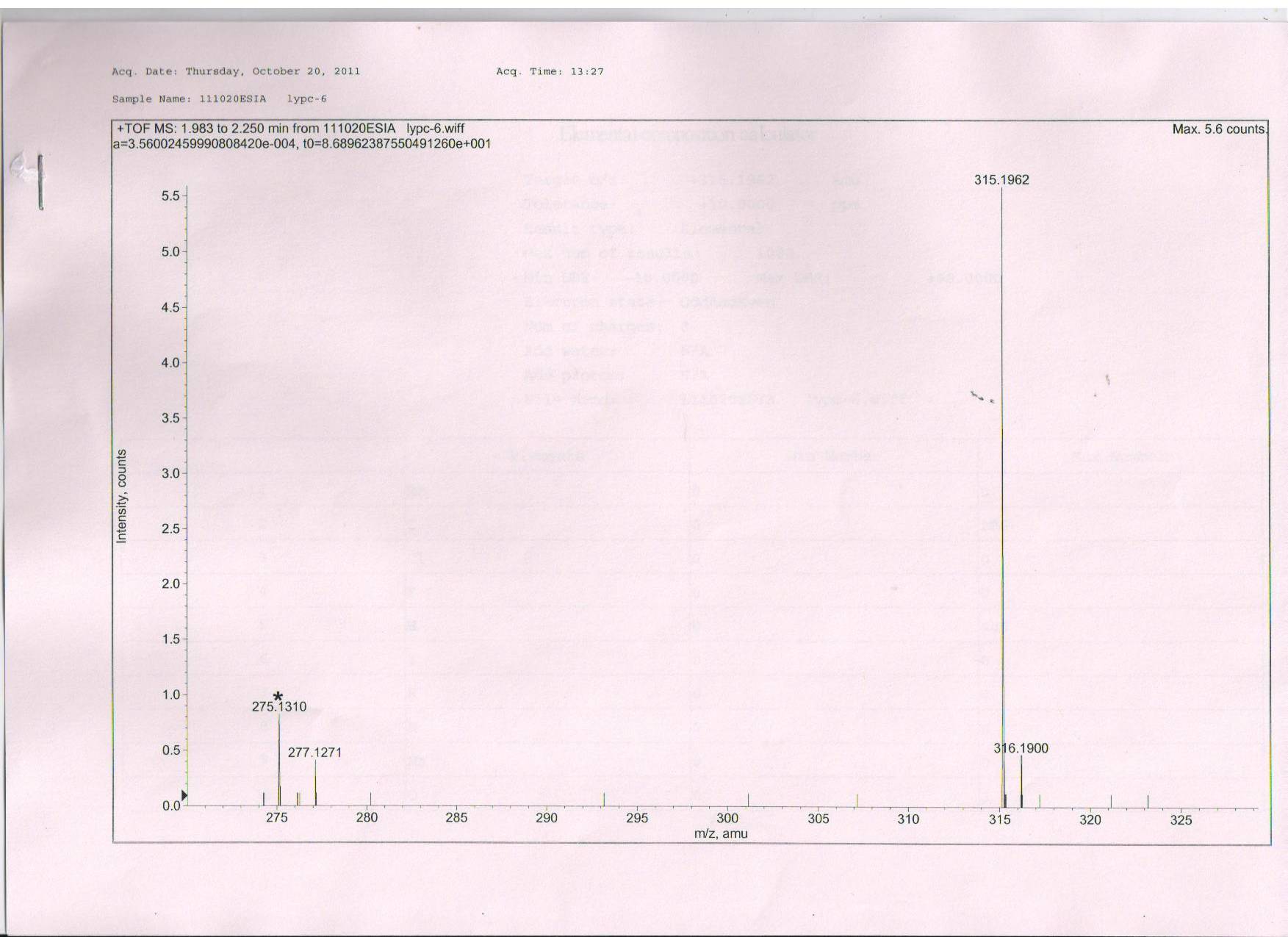


Figure 15S. ^1^H NMR of Guanacastepene R (**3**)


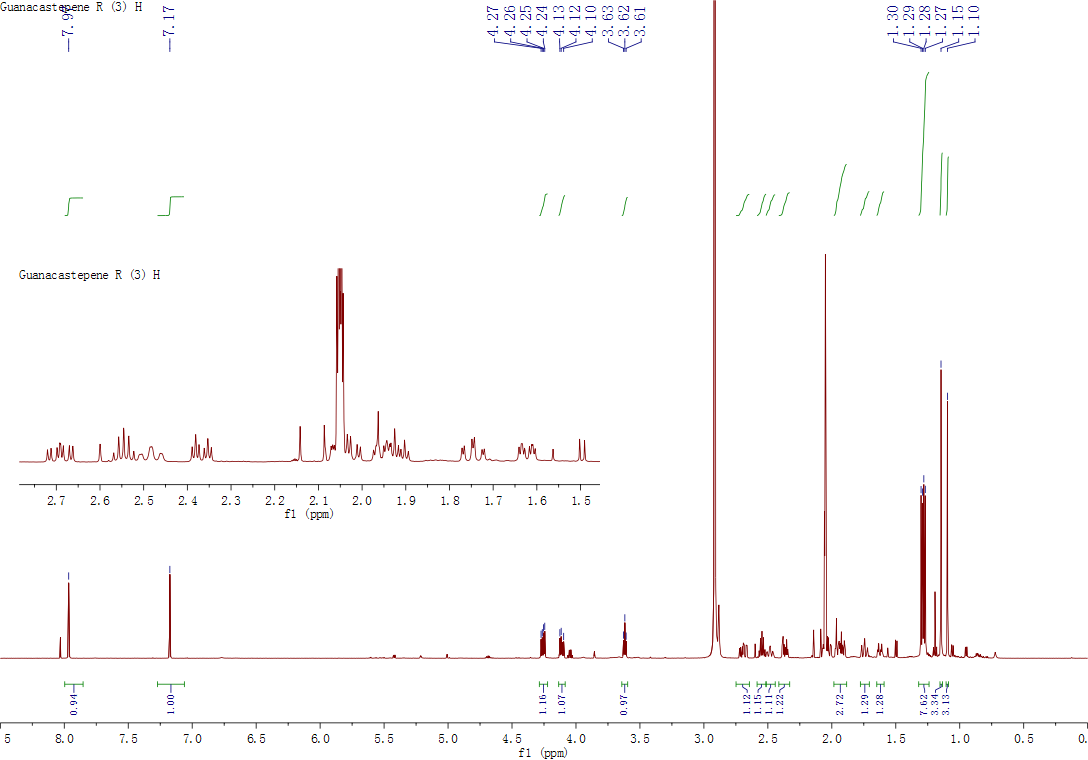


Figure 16S. ^13^C NMR and DEPT of Guanacastepene R (**3**)


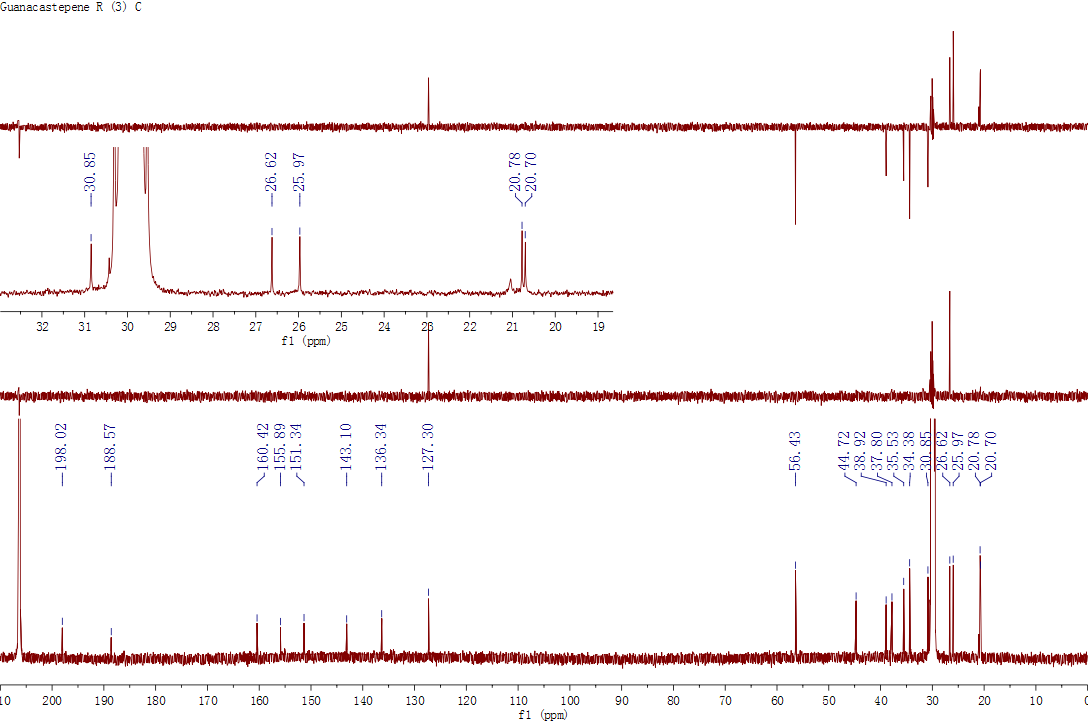


Figure 17S. HSQC of Guanacastepene R (**3**)


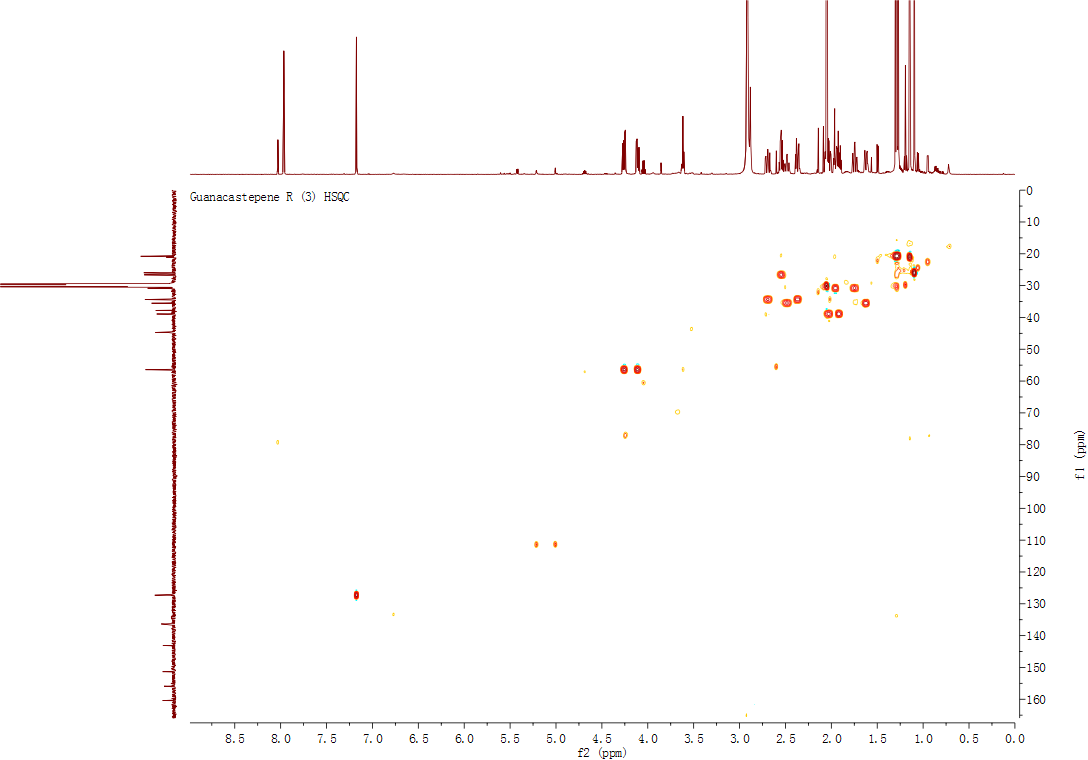


Figure 18S. HMBC of Guanacastepene R (**3**)


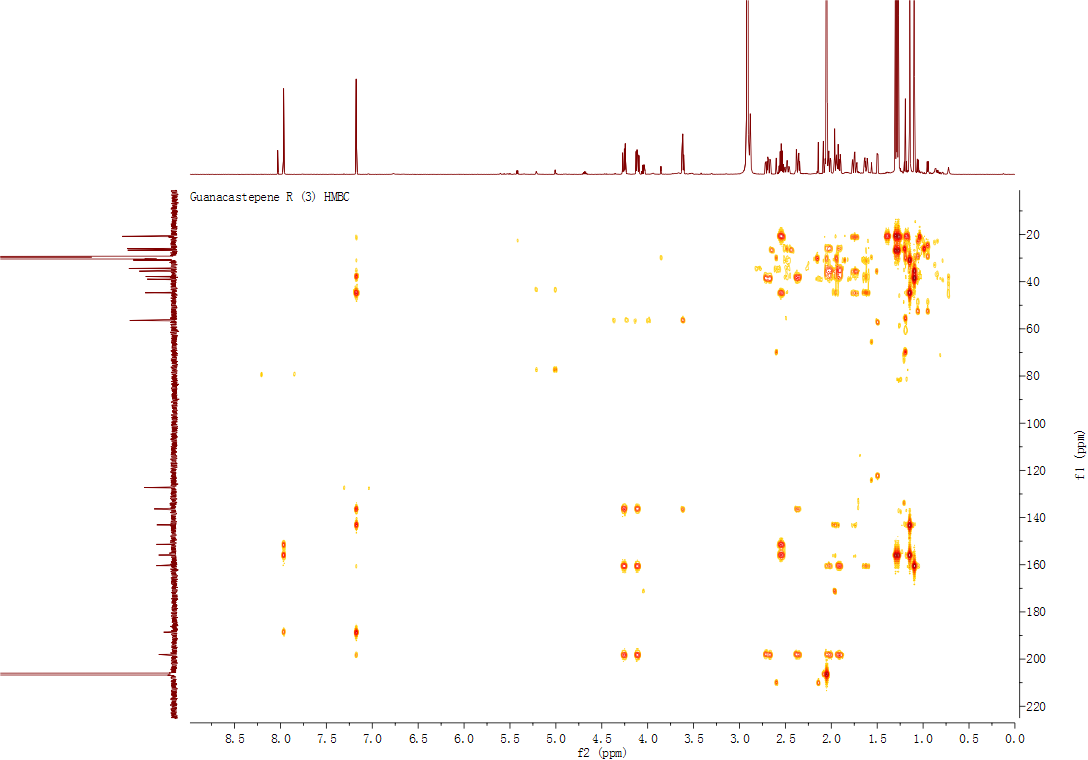


Figure 18΄S. enlarged HMBC of Guanacastepene R (**3**)


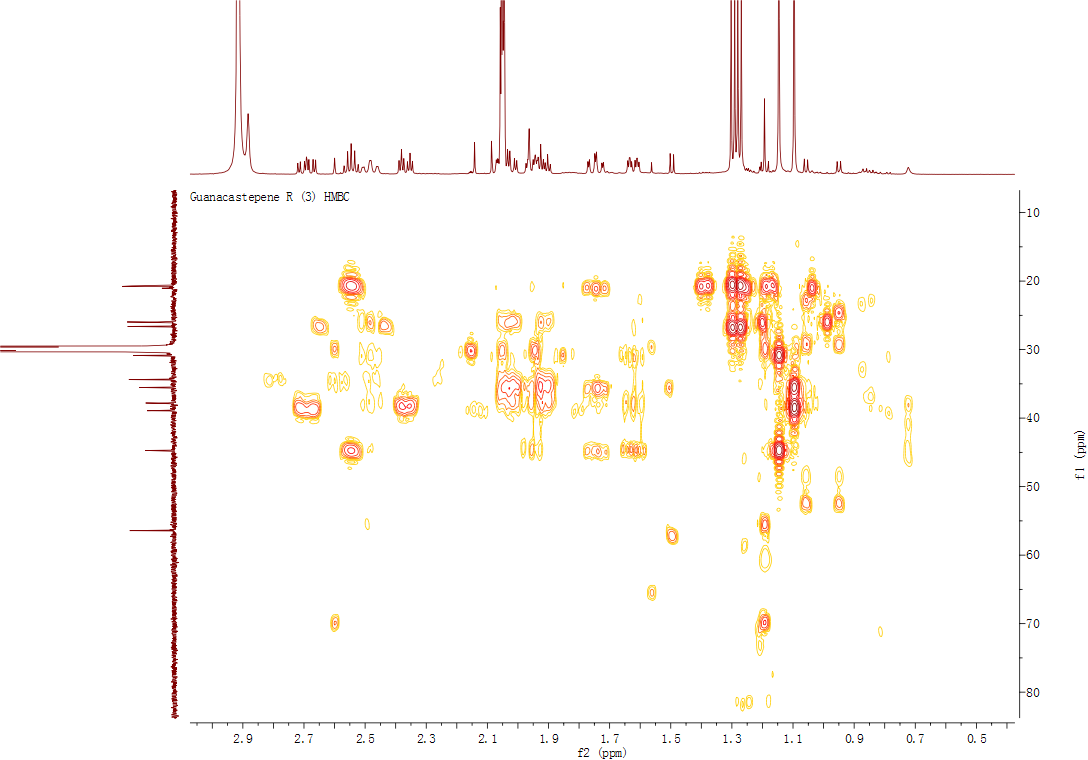


Figure 19S. ^1^H-^1^H COSY of Guanacastepene R (**3**)


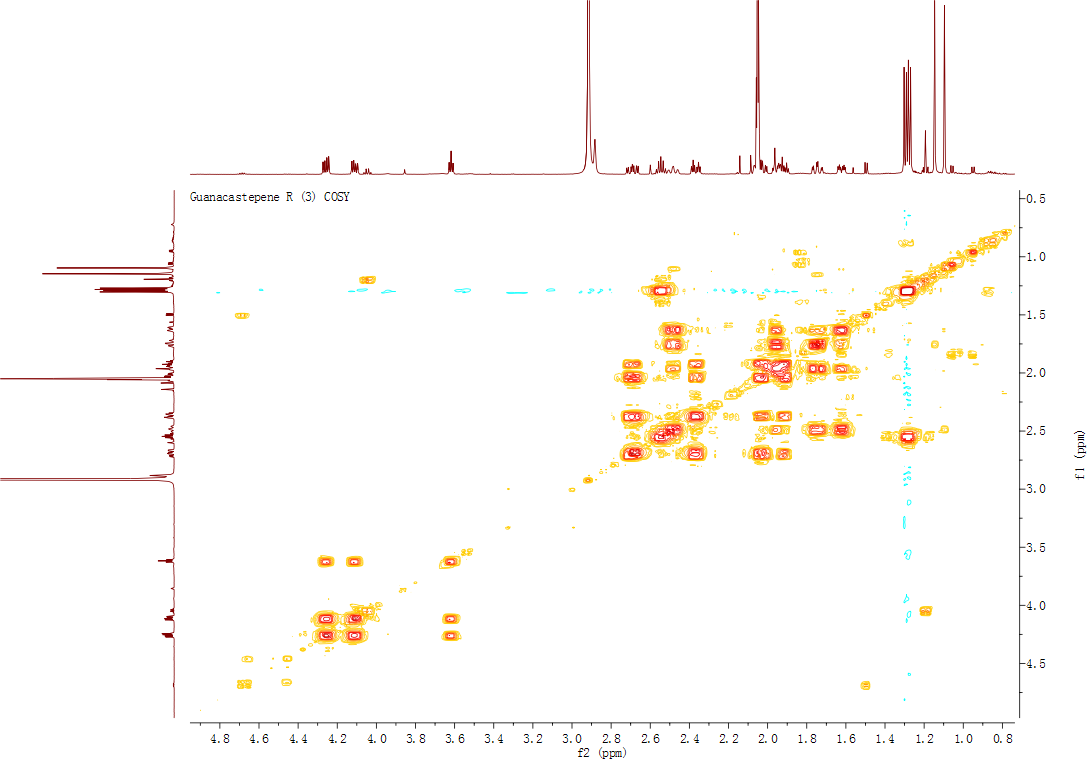


Figure 20S. ROESY of Guanacastepene R (**3**)


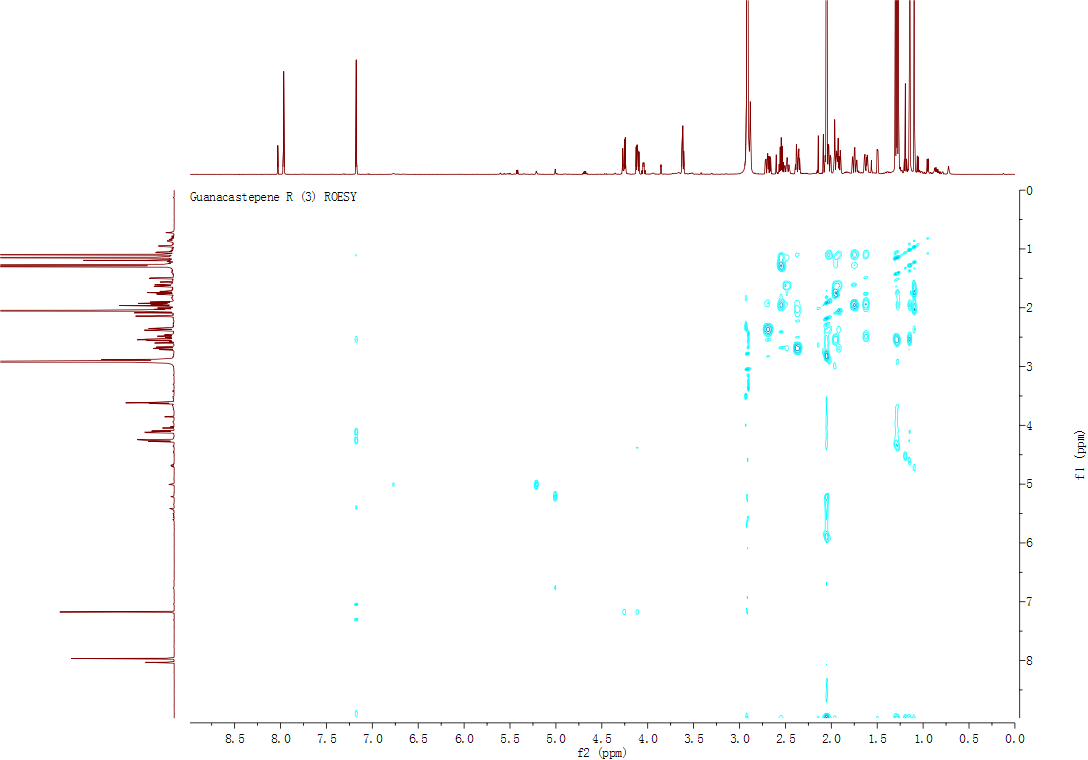


Figure 21S. HRESIMS of Guanacastepene R (**3**)


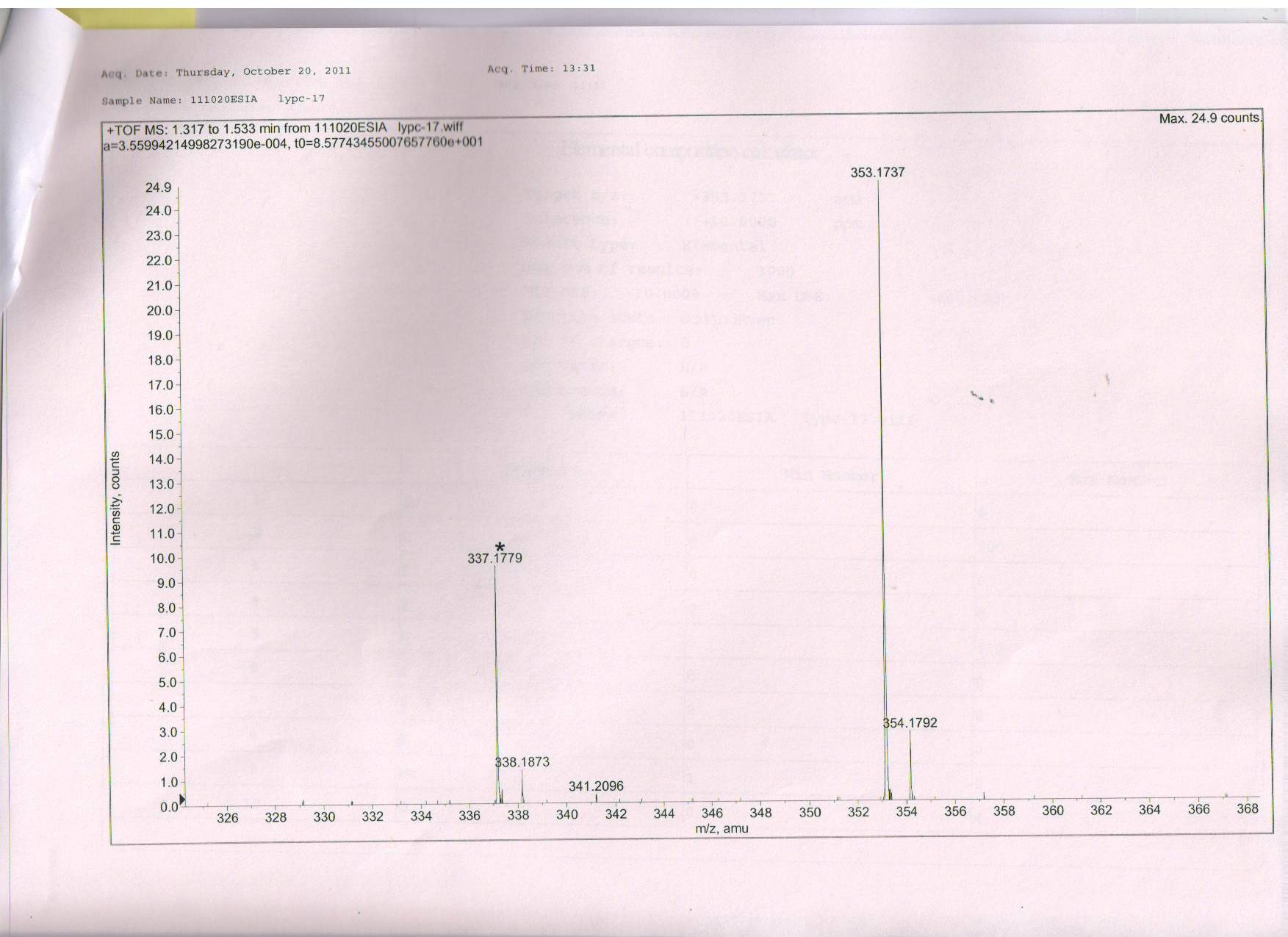


Figure 22S. ^1^H NMR of Guanacastepene S (**4**)


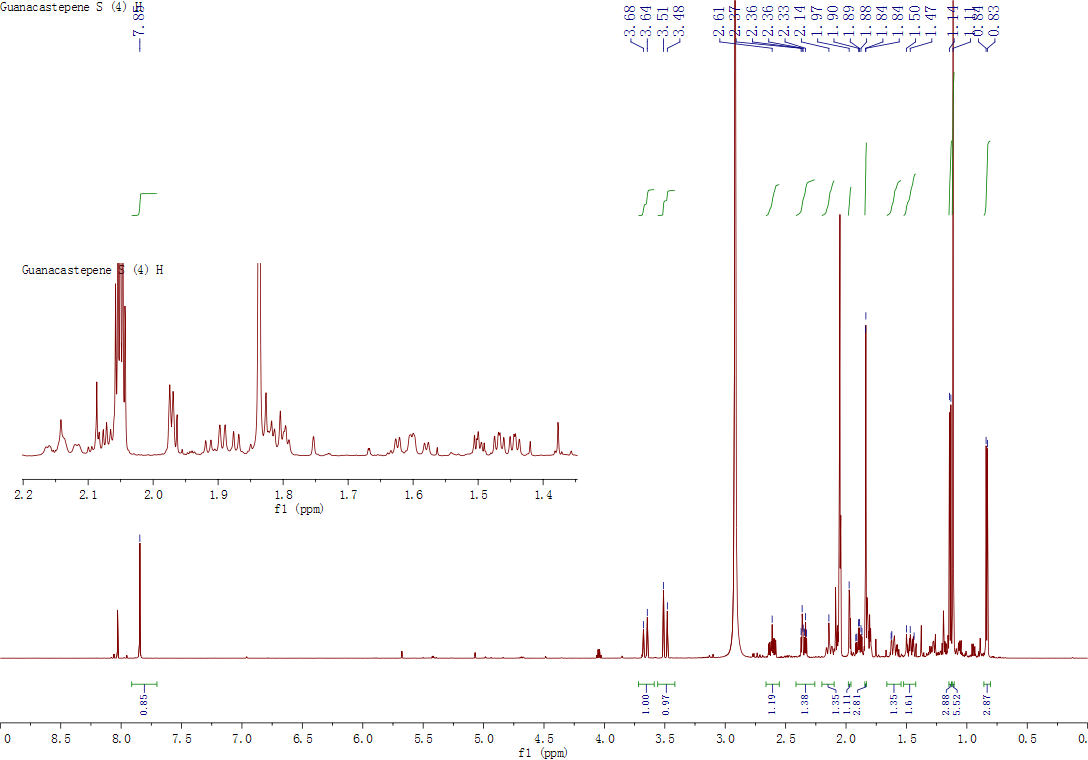


Figure 23S. ^13^C NMR and DEPT of Guanacastepene S (**4**)


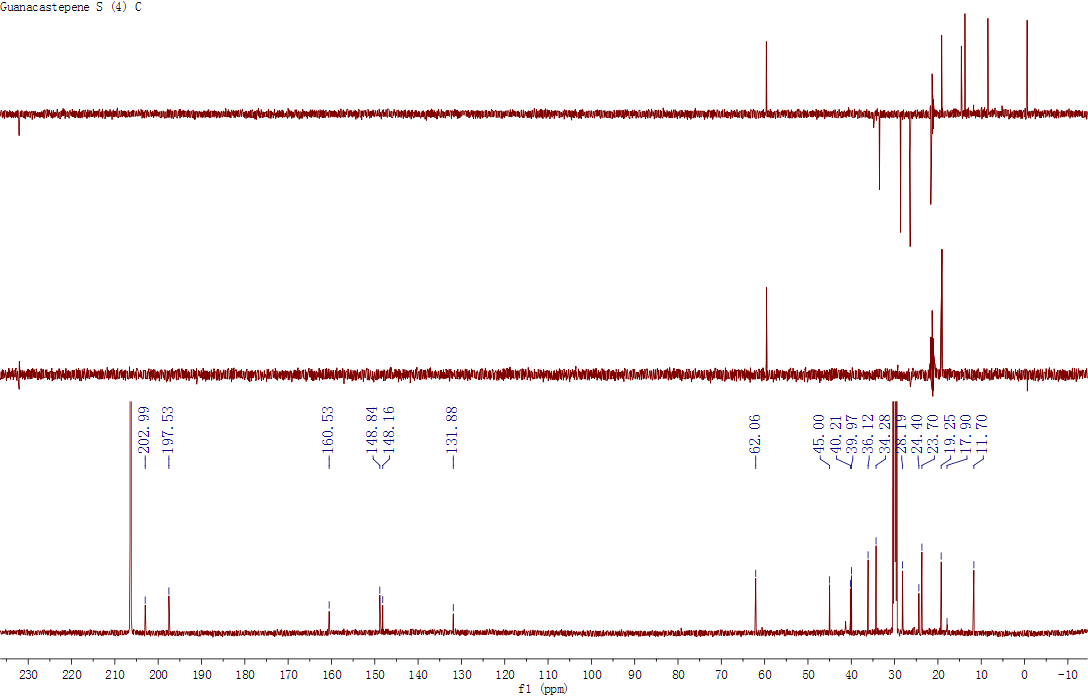


Figure 24S. HSQC of Guanacastepene S (**4**)


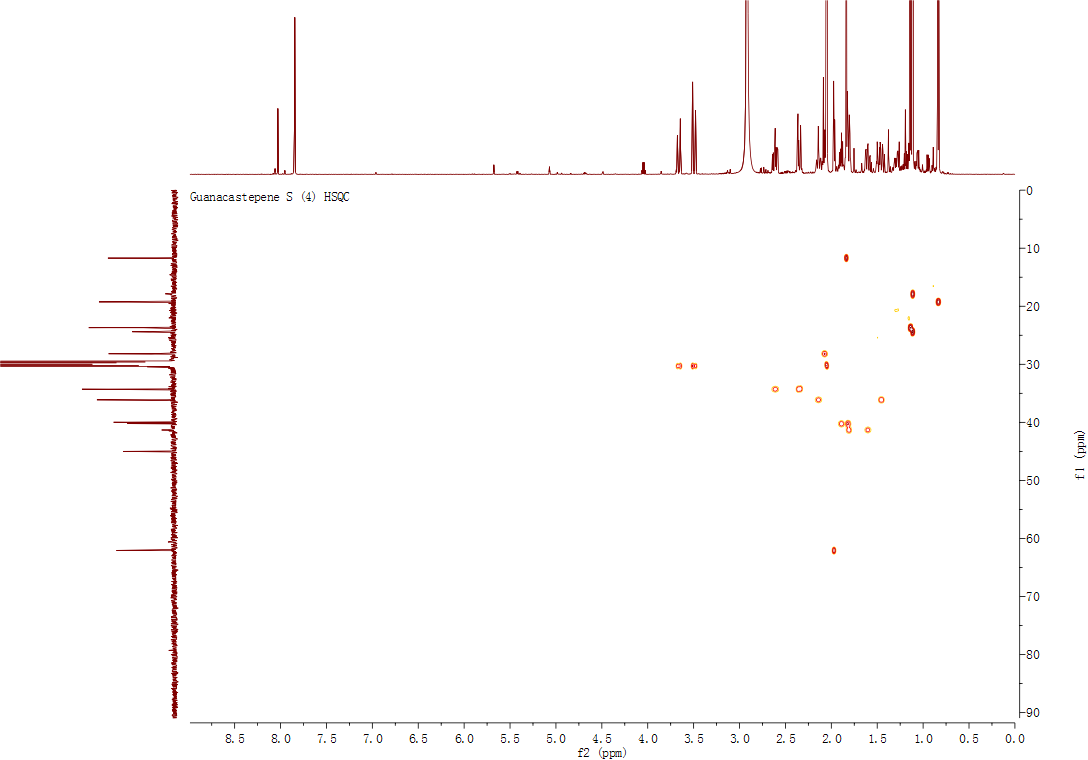


Figure 25S. HMBC of Guanacastepene S (**4**)


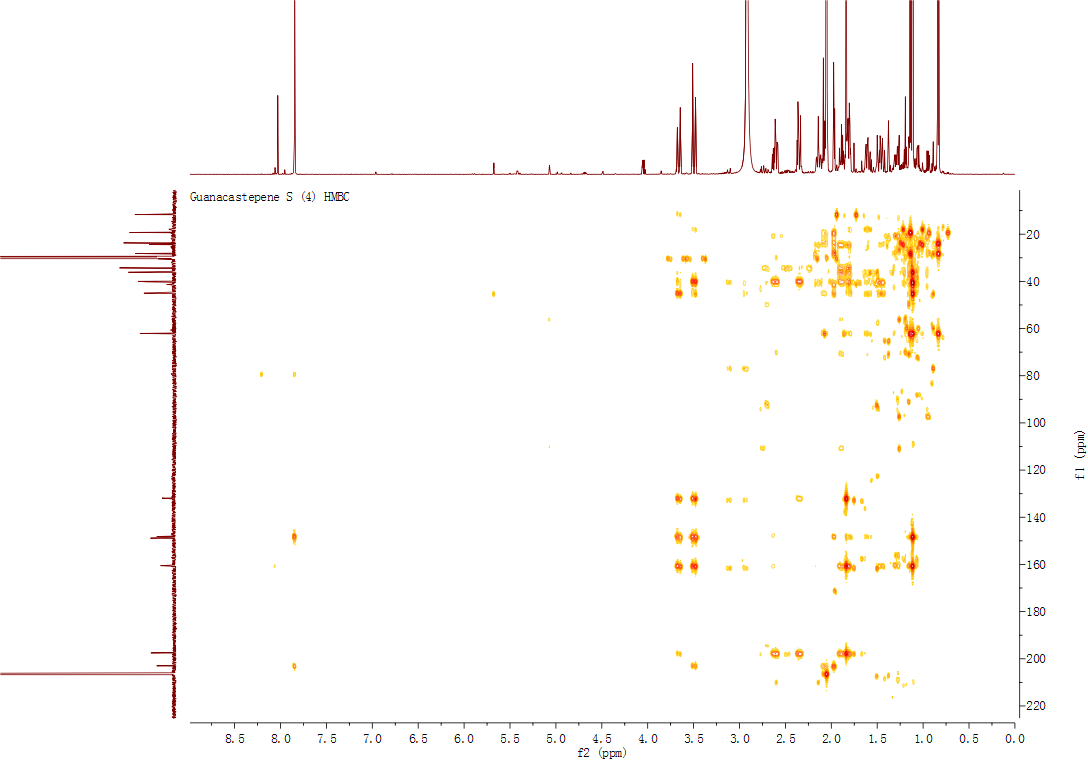


Figure 25΄S. enlarged HMBC of Guanacastepene S (**4**)


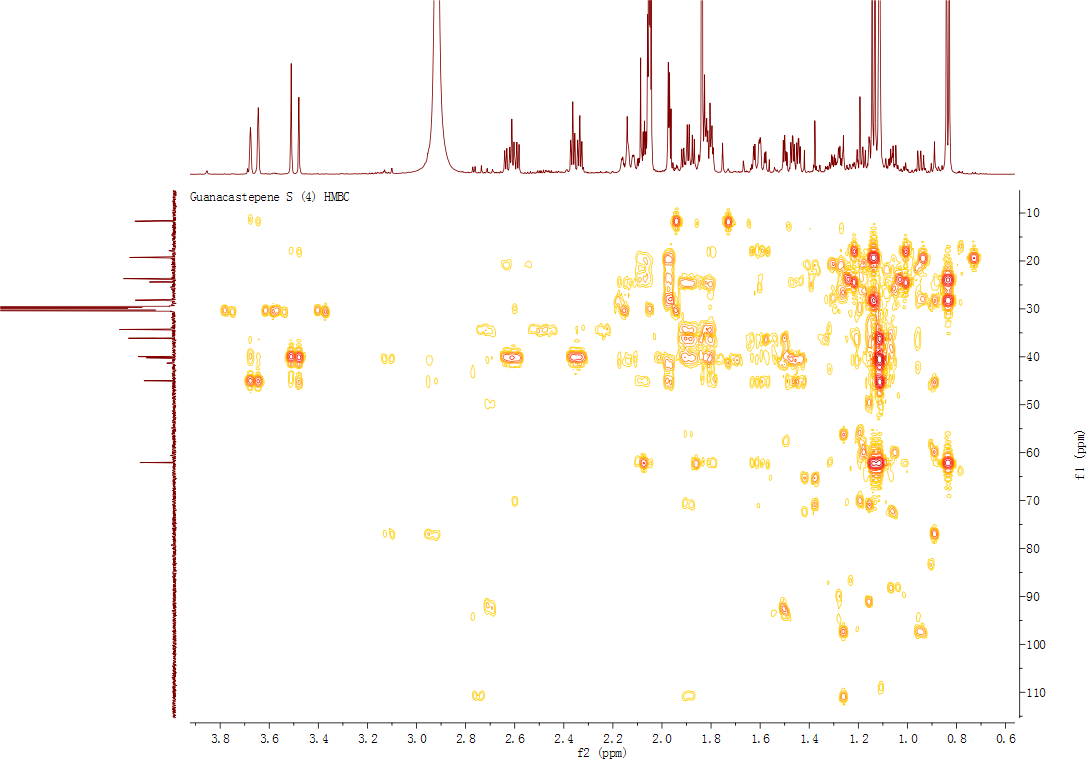


Figure 26S. ^1^H-^1^H COSY of Guanacastepene S (**4**)


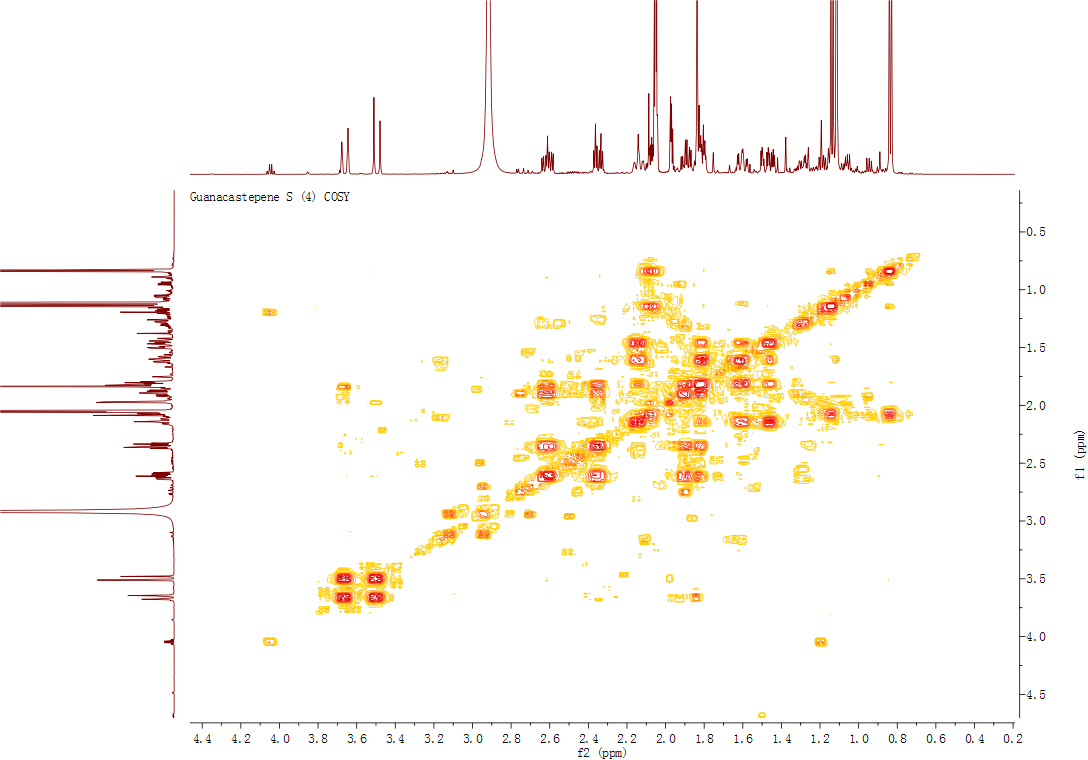


Figure 27S. ROESY of Guanacastepene S (**4**)


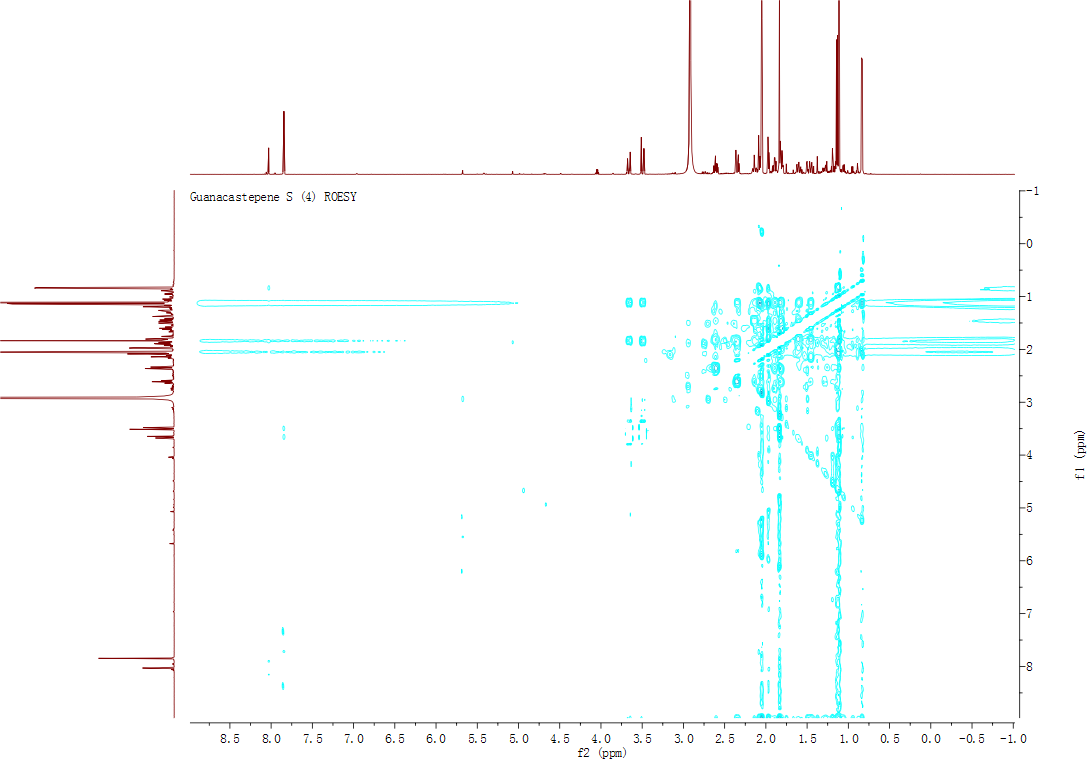


Figure 28S. HRESIMS of Guanacastepene S (**4**)


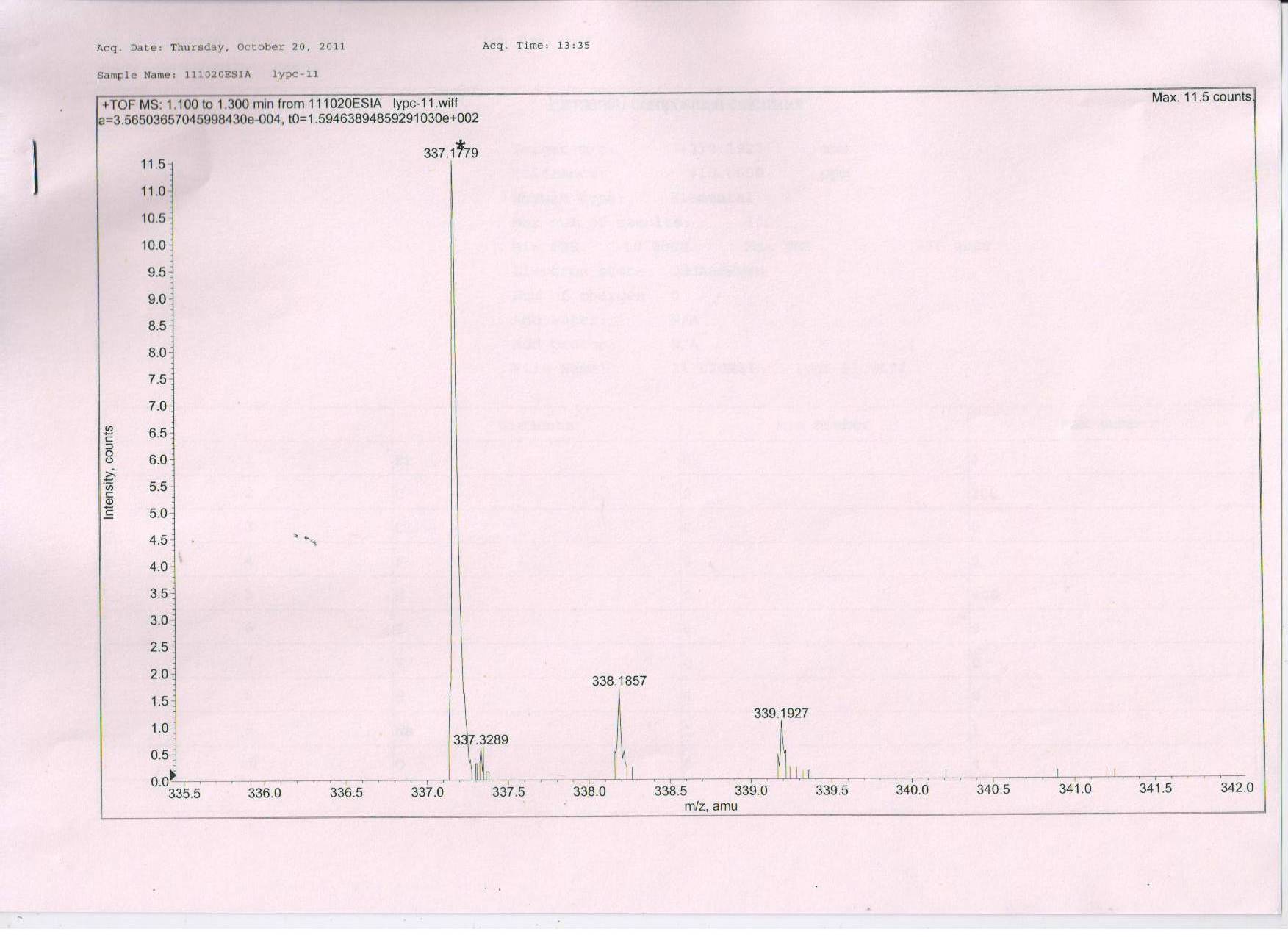


Figure 29S. ^1^H NMR of Guanacastepene T (**5**)


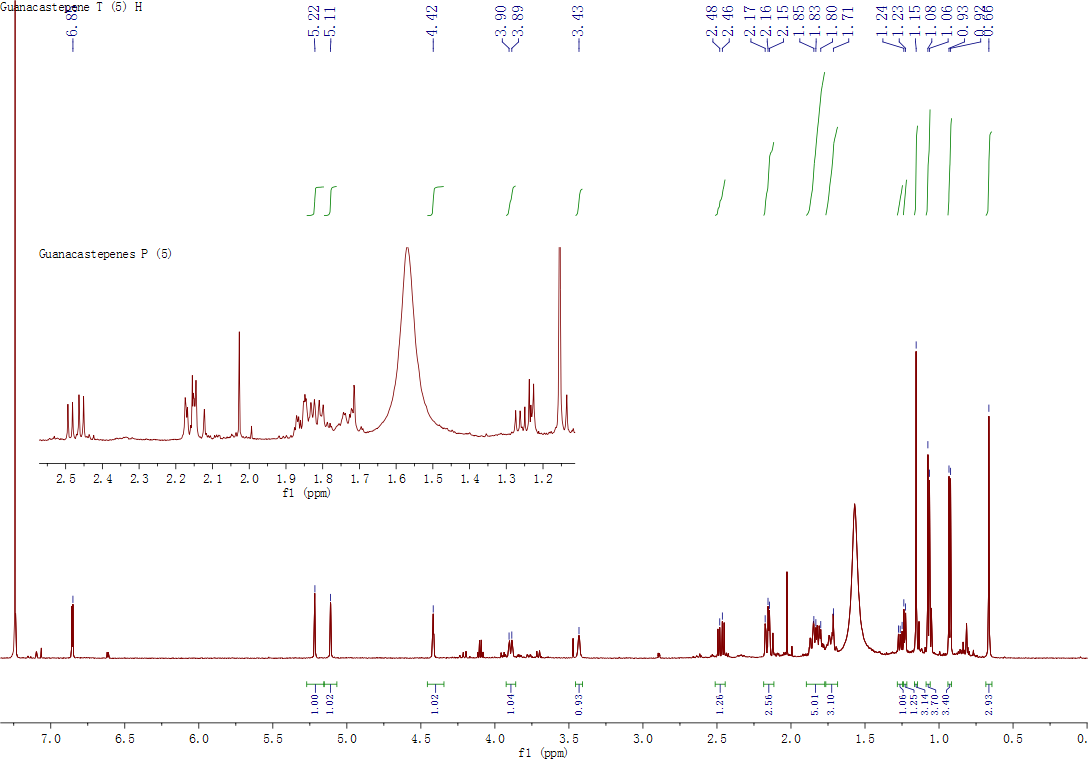


Figure 30S. ^13^C NMR and DEPT of Guanacastepene T (**5**)


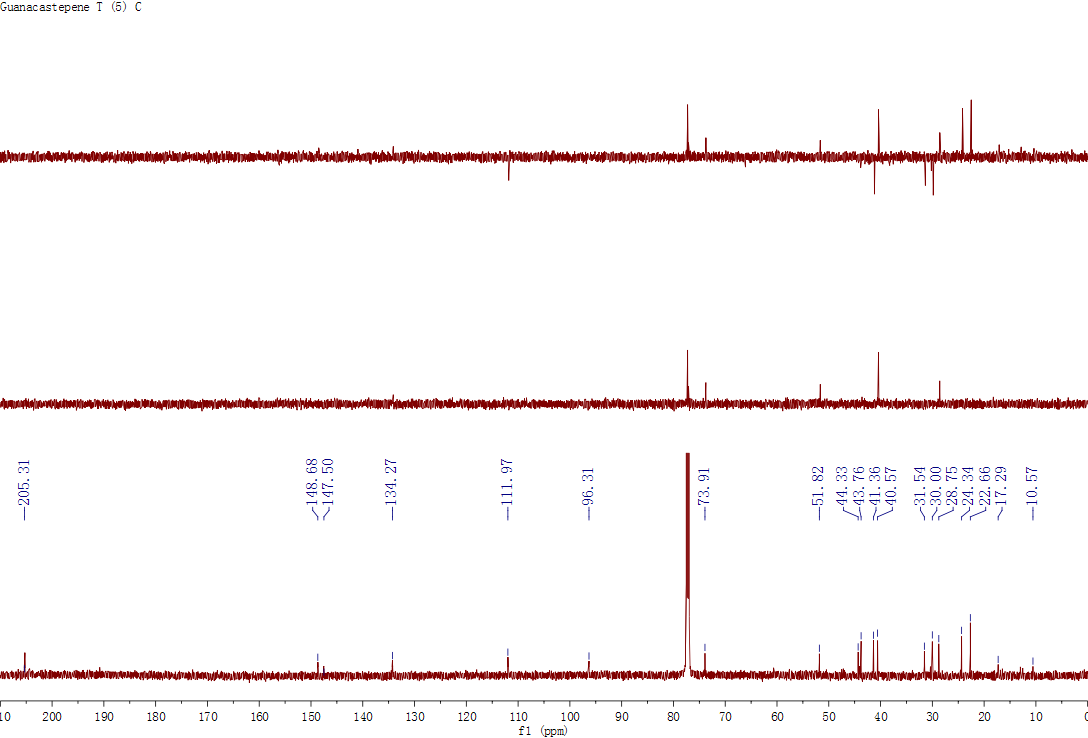


Figure 31S. HSQC of Guanacastepene T (**5**)


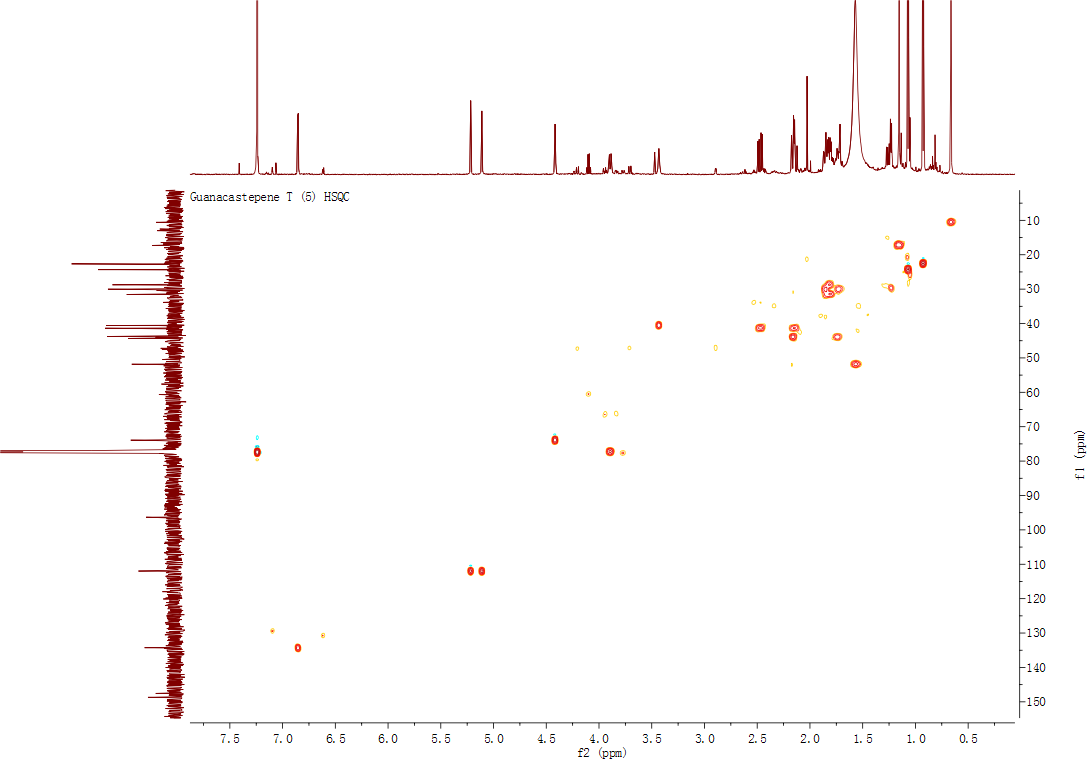


Figure 32S. HMBC of Guanacastepene T (**5**)


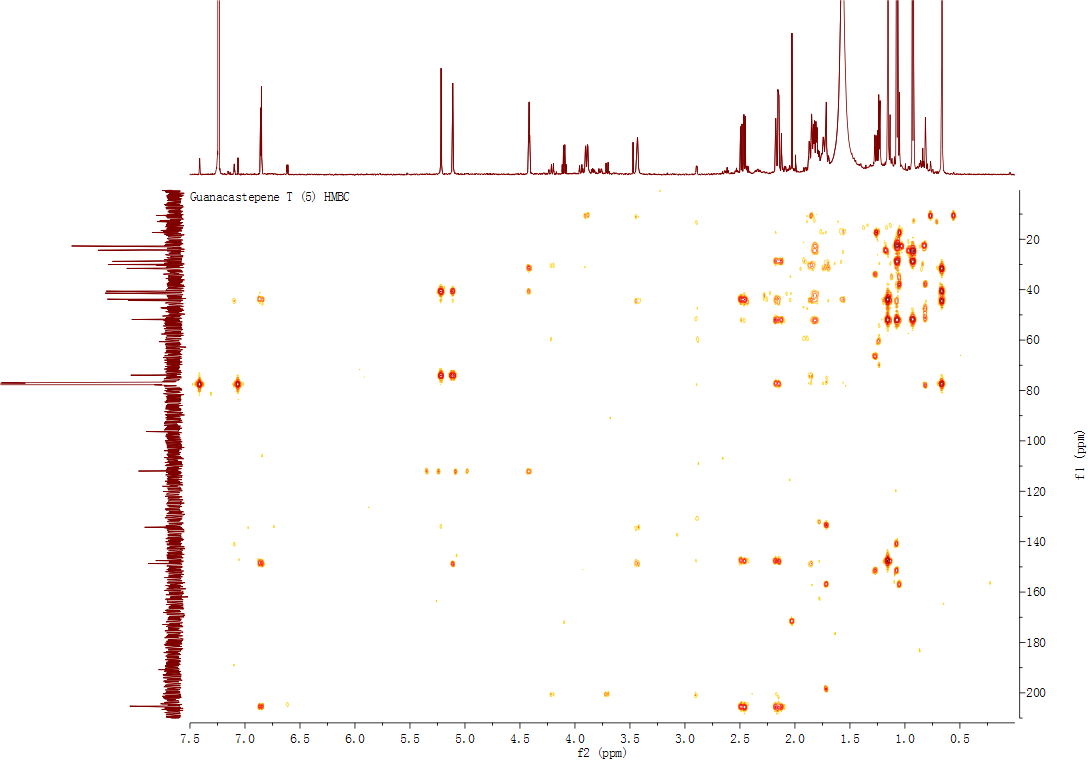


Figure 32΄S. enlarged HMBC of Guanacastepene T (**5**)


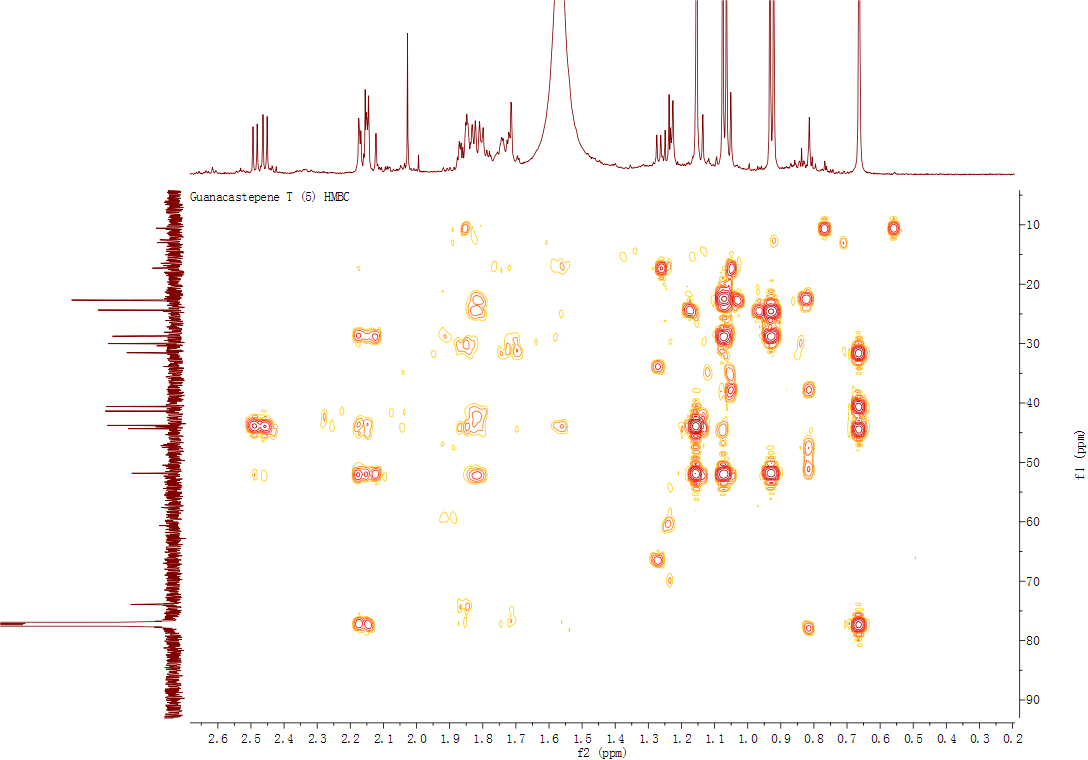


Figure 33S. ^1^H-^1^H COSY of Guanacastepene T (**5**)


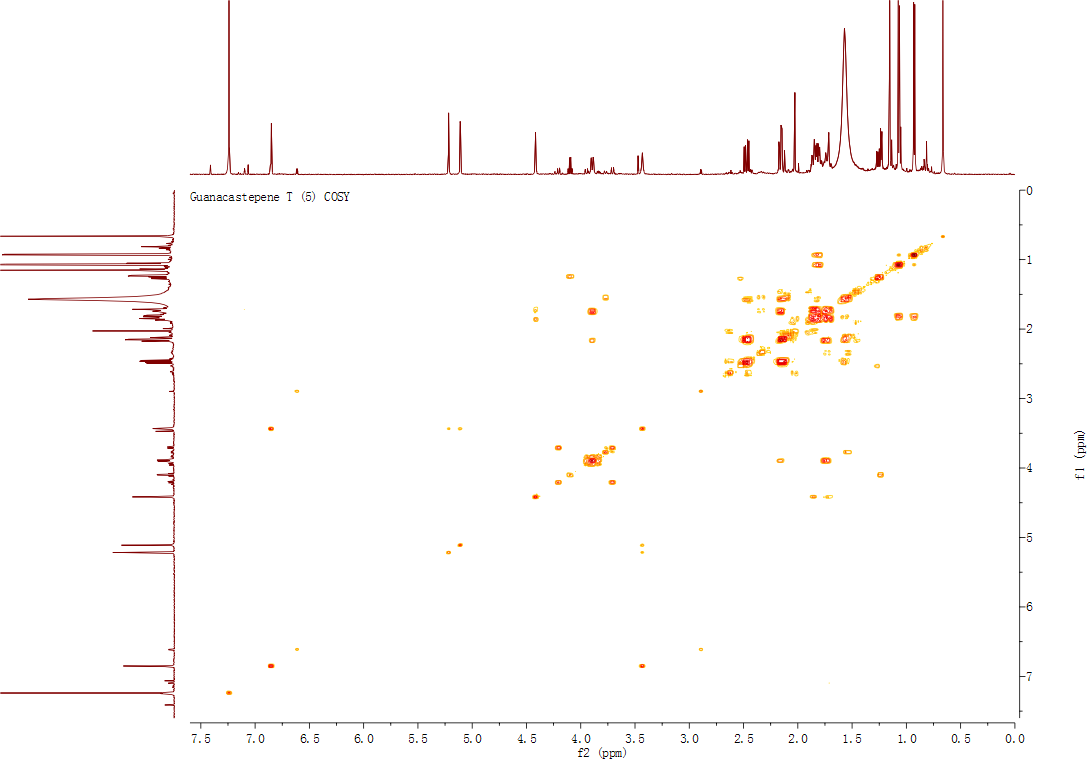


Figure 33΄S. enlarged ^1^H-^1^H COSY of Guanacastepene T (**5**)


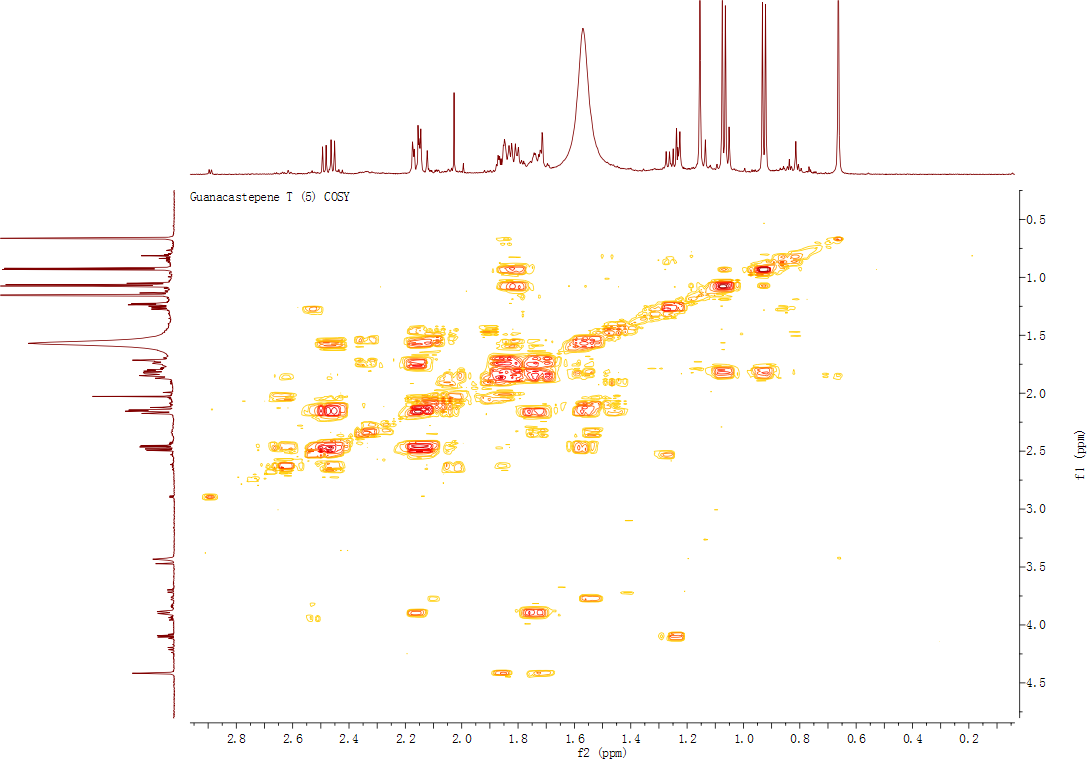


Figure 34S. ROESY of Guanacastepene T (**5**)


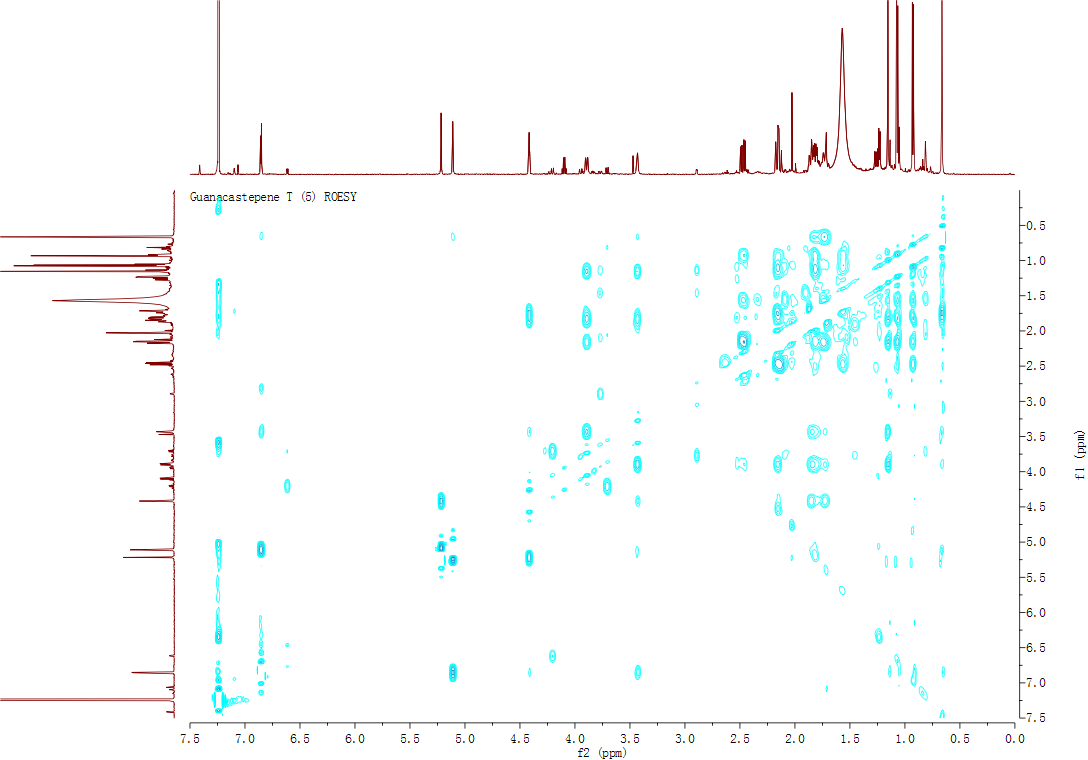


Figure 35S. HRESIMS of Guanacastepene T (**5**)


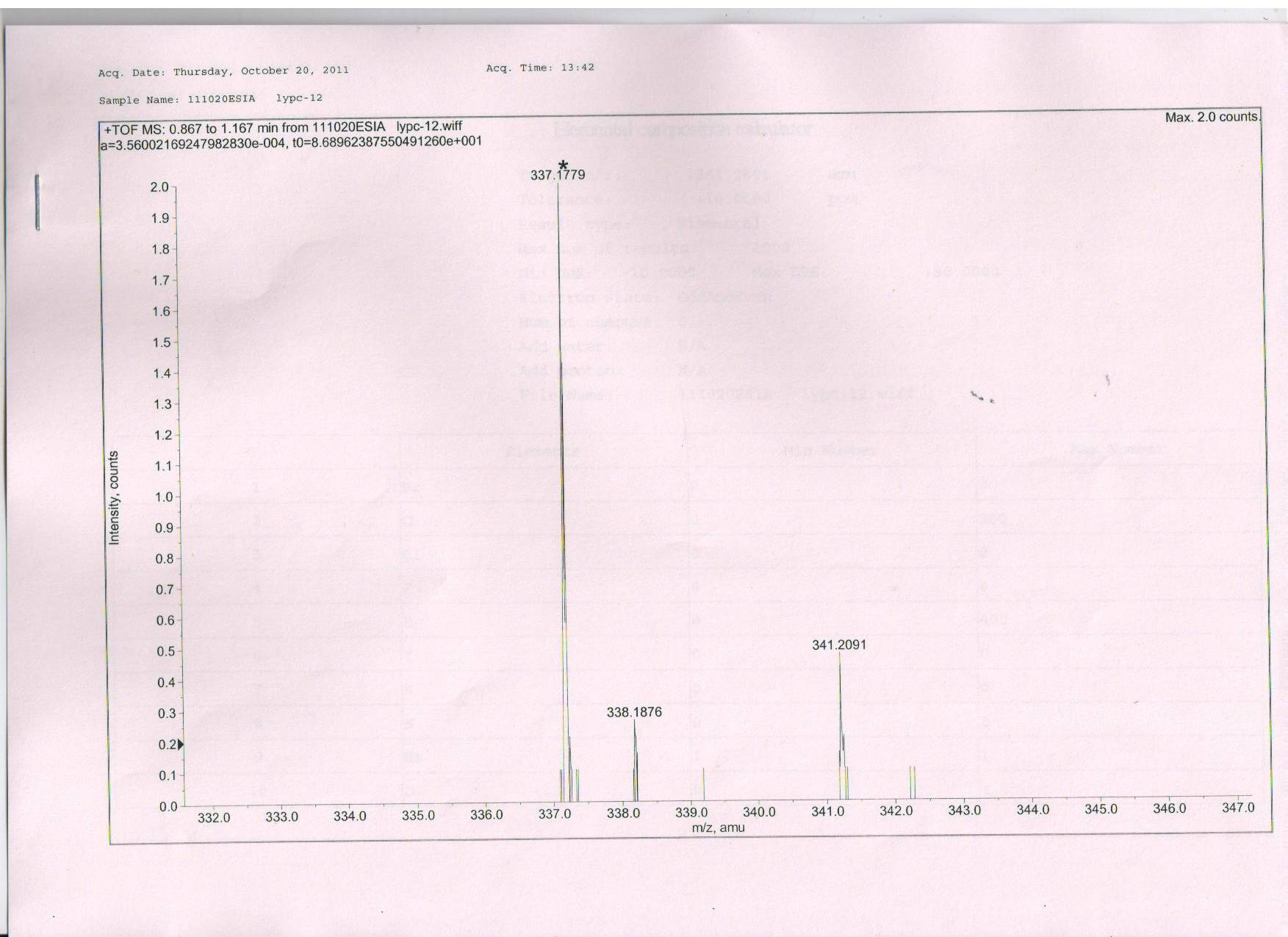

Supplement: Supplementary file 1 — Supplementary material 1 (DOC 4075 kb) [file 13659_2014_20_MOESM1_ESM.doc]
